# From Genome Inspection to Precision Agrochemicals: A Structure-Based Antivirulence Roadmap for Sustainable Crop Protection against *Xylella fastidiosa*

**DOI:** 10.1021/acs.jafc.5c16967

**Published:** 2026-05-26

**Authors:** Rosanna Caliandro, Andrea Astolfi, Serafina Serena Amoia, Benny Danilo Belviso, Vincenzo Mangini, Reinaldo Rodrigues De Souza Neto, Anna Giovanna Sciancalepore, Stefano Sabatini, Annalisa Giampetruzzi, Maria Letizia Barreca, Rocco Caliandro

**Affiliations:** † Institute for Sustainable Plant Protection, National Research Council (IPSP-CNR), via Amendola 122/D, 70126 Bari, Italy; ‡ Department of Pharmaceutical Sciences, University of Perugia, Via del Liceo 1, 06123 Perugia, Italy; § Institute of Crystallography, National Research Council (IC-CNR), via Amendola 122/o, 70126 Bari, Italy

**Keywords:** Xylella fastidiosa, virulence factors, protein structure determination, drug discovery, plant disease

## Abstract

The emergence of antimicrobial resistance and environmental concerns is driving the need for sustainable crop-protection strategies beyond conventional pathogen-killing approaches. Here, we outline a structure-based drug discovery (SBDD) framework tailored to *Xylella fastidiosa*, one of the most threatening bacterial plant pathogens worldwide. By adapting conceptual and methodological pillars from human drug discovery, our approach focuses on antivirulence strategies that target pathogenicity factors rather than on bacterial viability. We provide a rational discovery roadmap encompassing genomic analysis, target assessment, *in silico* identification of candidate agrochemicals, and experimental multilevel validation *in vitro* and *in planta*. Building on this, our paper offers both theoretical background and practical recommendations to accelerate the identification of safe and effective crop protectors against *Xylella fastidiosa*, ultimately supporting the development of more sustainable and evolutionarily robust plant-disease-management strategies.

## Graphical abstract

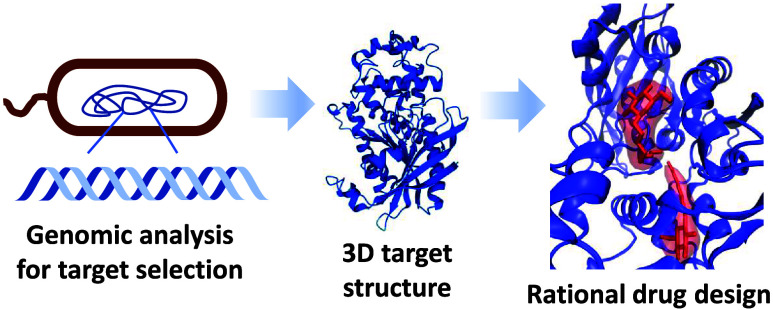

## 1 Introduction

*Xylella fastidiosa* is a xylem-limited, Gram-negative bacterium that has emerged as a major threat to global agriculture due to its high adaptability and broad host range, affecting numerous economically important crops including grapevine, citrus, stone fruits, olive, pecan, blueberry, alfalfa, and coffee as well as various ornamental and forest species such as elm, oak, sycamore, and oleander.^1,2^ First described in the early 20th century as the causal agent of Pierce’s Disease (PD) in grapevine,^3^ *X. fastidiosa* has since then been associated with a growing number of economically important plant diseases, including olive quick decline syndrome (OQDS), citrus variegated chlorosis (CVC), and almond leaf scorch. The pathogen is transmitted by xylem-feeding insect vectors, particularly sharpshooters and spittlebugs, which mediate its rapid spread across diverse agroecosystems.^4,5^

Since its first detection in Europe in 2013,^6^ *X. fastidiosa* has devastated olive orchards in southern Italy,^7^ and its continued spread poses a serious risk to various crops in the Mediterranean region and beyond. Its large genomic variability and the emergence of new genotypes (or sequence types) with different virulence traits and host range, and the ability to settle in new climate conditions, complicate the implementation of timely and effective containment strategies.

Current management practices mainly rely on vector control, eradication of infected hosts, and strict quarantine measures to prevent new introductions and limit spread.^7^ Other innovative emerging strategies include biological control,^8^ endophytic microbiota manipulation, application of antisense technologies based on small RNA interfering^9^ or bacteriophage-based treatments,^10^ although these latter remain at an experimental stage. The development of integrated pest management (IPM)^11^ frameworks combining vector suppression, host resistance, and environmental monitoring offers the most promising avenue for sustainable long-term control.

Currently, no control measures exist to completely eliminate *X. fastidiosa* from infected plants in the field.^12^ However, several promising preliminary studies have demonstrated a reduction in symptoms and bacterial populations within plants.^8,13^ It is worth emphasizing that each pathosystem requires a tailored control strategy.

The severe socio-economic impact of *X. fastidiosa* ^14^ and the limited efficacy of current control measures highlight the urgent need for innovative, effective strategies, supported by continued investment in multidisciplinary research.

In this Review, we discuss the potential of targeting bacterial virulence factors as a novel strategy for the sustainable and selective control of *X. fastidiosa*. By translating and contextualizing well-established structure-based drug discovery (SBDD) principles, widely used in human drug discovery, into the agrochemical domain, we provide an actionable roadmap for this pathogen, outlining inputs/outputs and practical decision points from genome-driven target selection to the functional validation of candidate small-molecule agrochemicals through *in vitro* and *in planta* assays (Figure 1).

**Figure 1:**
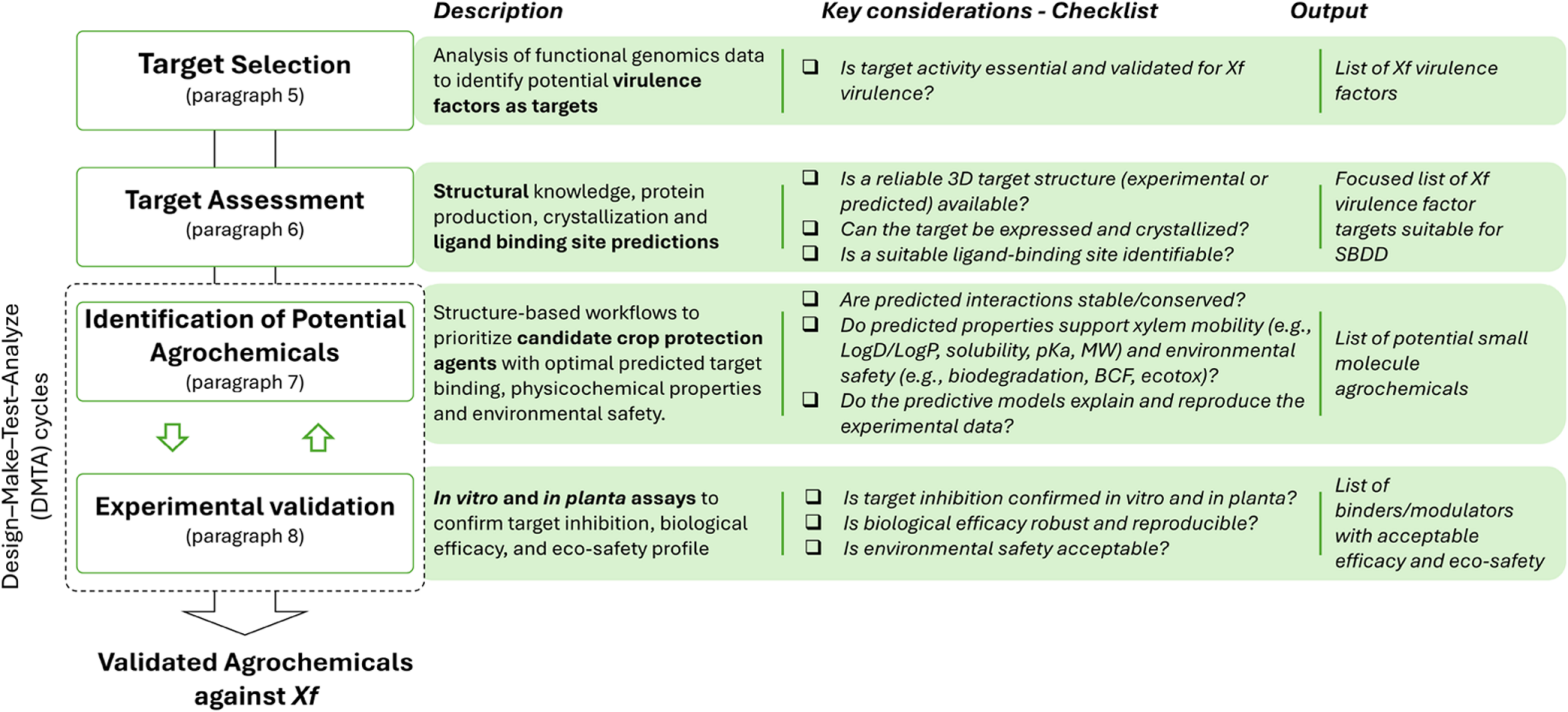
Schematic overview of a structure-based workflow followed to discover agrochemicals targeting *X. fastidiosa* virulence factors.

## 2 Comparative Genomics and Virulence Factors of *X. fastidiosa*

*X. fastidiosa* is the first plant pathogenic bacterium for which the complete genome sequence has been published by a consortium of researchers in Brazil.^15^ Since the release of this complete genome sequence, the need to devise new control strategies based on understanding pathogenicity has prompted several new sequencing projects. Particularly, until today, the number of genomes of *X. fastidiosa* publicly available is 270 (https://www.ncbi.nlm.nih.gov/datasets/genome/?taxon=2371 accessed in November 2025).

Comparison of genome sequences has led to the identification of genes that are conserved among virulent strains causing infection in a specific host but are less pathogenic in other ones, potentially leading to the discovery of new specific virulence factors. In Dow et al.,^16^ the authors highlighted some of the similarities (and differences) between gene products and gene organization in *Xanthomonas* and *Xylella* that may enlarge studies of *X. fastidiosa* pathogenesis. Uceda-Campos et al.^17^ further expanded these insights through a comprehensive comparison of American and European isolates, uncovering patterns of gene loss and acquisition linked to niche adaptation, particularly affecting adhesins and surface-exposed proteins. Similarly, Denancé et al.^18^ analyzed French isolates of *X. fastidiosa*, revealing genome plasticity that likely facilitates host range expansion and environmental adaptation.

Further advances in comparative genomics have revealed that both inter- and intrasubspecific homologous recombinations significantly contribute to the diversification of *X. fastidiosa* virulence factors. Potnis et al.^19^ demonstrated that genes associated with adhesion, biofilm formation, and host interaction are frequent targets of recombination events, thereby promoting eco-evolutionary adaptability.

Collectively, these findings highlight the pivotal role of genomic plasticity in shaping *X. fastidiosa* pathogenicity and underscore the relevance of conserved virulence determinants as promising molecular targets for innovative crop-protection strategies.

## 3 Antivirulence Approaches: A Paradigm Shift in Plant Protection

In recent years, research and development efforts in the antimicrobial field have increasingly turned to alternative strategies that target bacterial pathogens through selective inhibition of virulence factors rather than pursuing traditional bactericidal approaches, which often suffer from nonspecific toxicity and the risk of selecting for resistance.^20^

The rationale behind the antivirulence strategy is to combat the rise of antimicrobial resistance by disarming pathogens rather than directly killing them. Small-molecule-based agrochemicals designed to disrupt bacterial virulence mechanisms—essential for host colonization and pathogenesis but not for bacterial survival—can mitigate disease progression by reducing host damage and providing the host’s immune system sufficient time to clear the pathogen, while sparing beneficial microbiota and thereby enhancing ecological competition that further disadvantages pathogenic bacteria.^21–23^ Importantly, in the context of *X. fastidiosa*, targeting conserved virulence determinants for the development of sustainable and precision agrochemicals dedicated to crop protection could ensure broad-spectrum efficacy across its diverse strains and subspecies.

Building on these insights, we delineate how such macromolecular targets can be rationally leveraged within a modern SBDD-guided discovery framework to identify effective antivirulence candidates for agricultural use.

## 4 Structure-Based Drug Discovery (SBDD) as a Framework for Precision Agrochemicals

To translate virulence factor inhibition into tangible crop protection tools for *X. fastidiosa*, we discuss the adaptation of the classical SBDD pipeline well established in human therapeutics^24–26^ and increasingly adopted in the agrochemical industry^27–32^ to the development of precision agrochemicals specifically directed against this challenging bacterium and designed to minimize environmental and toxicological impacts. In this manuscript, we focus on a computational-first SBDD framework, in which target assessment and hit identification/prioritization are performed primarily *in silico*, and no experimental screening (*e.g.*, X-ray fragment screening) is included at this early discovery stage.

In this context, SBDD leverages detailed three-dimensional (3D) structural data on biological targets to guide the identification and optimization of novel therapeutic agents. Unlike traditional high-throughput screening, this rational approach focuses on understanding the specific interactions between molecules and their biological targets, enhancing the success rates and overall efficiency in drug discovery.

In human medicine, SBDD has revolutionized targeted therapies for diseases like cancer, infections, autoimmune disorders, and neurodegeneration, significantly reducing timelines and costs^24^ and is emerging as a promising strategy for rational design against molecular targets in pests and pathogens; however, its literature base and methodological standardization remain comparatively limited when compared to human therapeutics. This precision targeting enhances selectivity, reducing harm to beneficial organisms, cultivated plants, and soil microbiota, and minimizes environmental impact through lower application rates and reduced chemical runoff. Additionally, targeted mechanisms reduce pesticide resistance risks, thereby positioning SBDD as a powerful approach for developing precision agrochemicals against challenging pathogens like *X. fastidiosa* and ultimately supporting sustainable crop protection and global food security. Notably, the potential of this strategy is exemplified by the recently reported BASF SE’s mesoionic insecticide Fenmezoditiaz, which demonstrates how SBDD can be successfully applied to agrochemical development.^33^

The SBDD pipeline involves selecting and structurally characterizing a biological target, typically using techniques such as X-ray crystallography, NMR spectroscopy, or cryo-electron microscopy. When experimental data are unavailable, computational models (*e.g*., homology- or AI-predicted structures) offer viable alternatives. Structural analysis identifies ligand binding sites and key residues involved in molecular interactions. Computational chemistry and molecular modeling are then employed to design small-molecule ligands with potentially high affinity and selectivity. Predicted candidates undergo rigorous experimental validation, including chemical synthesis and biological testing, to assess efficacy and safety.

Within the framework of antivirulence approaches, we propose a systems-level rational drug discovery strategy that encompasses the following steps (Figure 1):

- Target Selection: Genomic and Functional Genomic Insights into *X. fastidiosa* Virulence Factors (Paragraph 5).
- Target assessment: structural knowledge, protein production and crystallization, ligand-binding-site predictions (paragraph 6).
- *In Silico* Identification of Potential Agrochemicals (Paragraph 7).
- Experimental validation: multilevel biological assays for *in vitro* and *in planta* studies on candidate agrochemicals (Paragraph 8).

In the following sections, we detail how these established steps can be operationalized for *X. fastidiosa* antivirulence targets, highlighting which elements are essential and which can be adapted depending on the available resources and the intended application context.

## 5 Target Selection: Genomic and Functional Genomic Insights into *X. fastidiosa* Virulence Factors

Functional genomics approaches such as gene knockouts, RNA interference (RNAi), transposon mutagenesis (Tn-seq), and transcriptomics have enabled the experimental validation of virulence-associated genes. Building on an extensive survey of the available literature, we systematically compiled and critically evaluated most of the virulence factors reported for *X. fastidiosa*. Table 1 lists the identified set of experimentally validated virulence determinants, derived from independent studies in which knockout mutants were assessed for bacterial pathogenicity *in planta*. Their roles in the biology of the pathogen are outlined in Figure 2. In the following sections, each virulence factor will be described in the context of *X. fastidiosa* metabolism.

**Figure 2:**
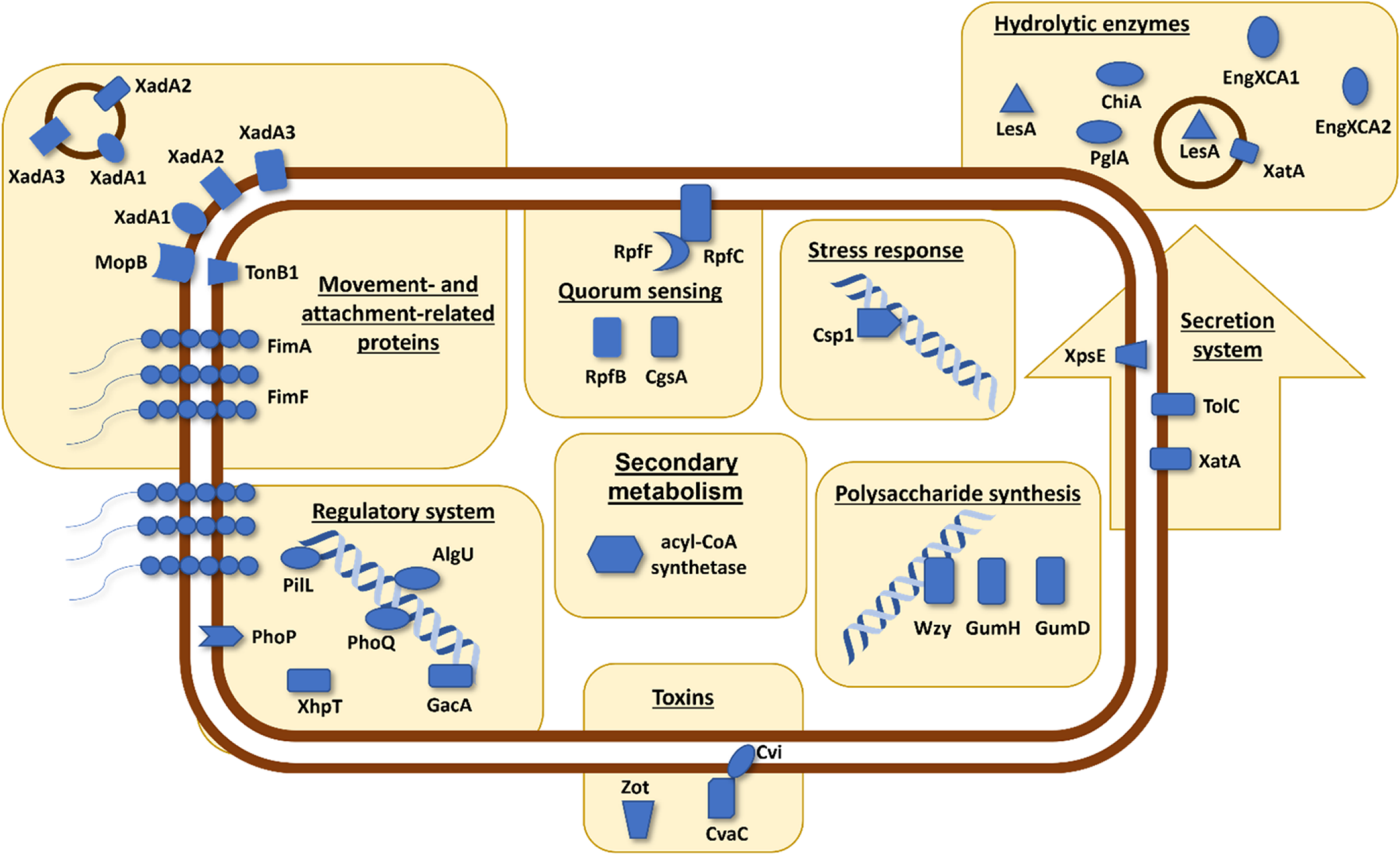
Outline of the main virulence factors of *X. fastidiosa* involved in the biology and pathogenicity of the pathogen.

**Table 1:** List of Virulence/Pathogenicity Factors of *X. fastidiosa* ^a^

gene	UNIPROT ID	function	mutant phenotype in virulence/pathogenicity
Secretion Systems			
*tolC*	Q87A67	Type I secretion system	Loss
*xpsE*	Q87DF3	Type II secretion system: ATPase	Loss
*xatA*	Q87E01	Type V secretion system	Reduced
Hydrolytic Enzymes			
*lesA*	Q87AW0	Secreted lipase/esterase	Loss
*engXCA1*	Q87AG9	Endoglucanase: pit membrane degradation	Reduced
*engXCA2*	Q87AH4	Endoglucanase: pit membrane degradation	Reduced
*pglA*	Q878G8	Polygalacturonase: pit membrane degradation	Loss
*chiA*	Q87AJ8	Chitinase	Loss
Quorum Sensing			
*rpfF*	Q87AI5	DSF synthesis	Enhanced, but impairment in vector transmission
*rpfC*	Q87EB1	Signal transduction	Reduced
*cgsA*	Q87EM4	Cyclic di-GMP synthase A	Reduced
*rpfB*	Q87ER5	DSF processing	Reduced
Regulatory Systems			
*algU*	Q87C12	Regulatory gene: control of virulence and pathogenicity genes	Reduced
*pilL*	Q87D38	Twitching mobility	Reduced
*phoQ*	Q87AY5	Two-component regulatory system: pathogenicity-related genes, survival genes	Loss
*phoP*	Q87AY4	Two-component regulatory system: pathogenicity-related genes, survival genes	Loss
Regulatory Systems			
*gacA*	Q87A47	Two-component regulatory system: pathogenicity-related genes	Reduced
*xhpT*	Q87BR2	Regulation of biofilm formation genes and EPS production	Reduced
Polysaccharide Synthesis			
*gumD*	Q87BQ4	Exopolysaccharide synthesis	Loss
*gumH*	Q87BQ7	Exopolysaccharide synthesis	Loss
*wzy*	Q87D72	Lipopolysaccharide synthesis	Reduced
Movement and Attachment			
*fimA*	Q87F75	Fimbrial adhesin: cell–cell aggregation, biofilm formation	Reduced
*fimF*	Q87F78	Fimbrial adhesin: cell–cell aggregation, biofilm formation	Reduced
*xadA1*	Q87DF4	Adhesin	Reduced
*xadA2*	Q9PD50	Adhesin	Reduced
*xadA3*	Q87D62	Adhesin	Enhanced, but impairment in vector transmission
*mopB*	Q87AV4	Required for bacterial type IV pili biogenesis and twitching motility	Reduced
*tonB1*	Q87D43	Iron and vitamin B12 transport	Reduced
Toxins			
*zot*	Q877P5	Putative Zot-like toxin	Reduced
*cvaC/cvi*	Q9PGN6/7	Microcin cvaC like/immunity protein	Reduced
Stress Response			
*csp1*	Q87BR8	Cold shock protein	Reduced
Secondary Metabolism			
PD_1311	Q87BY5	Acyl-coenzyme A (acyl-CoA) synthetase	Loss

a Adapted from Rapicavoli et al.^2^. The name of the gene, the ID number in Uniprot database, and its function are also reported.

### 5.1 Secretion System

The gene *tolC* encodes for a periplasm/outer membrane protein belonging to the type I secretion system, and it is highly conserved among many phytopathogenic bacteria.^34^ The *X. fastidiosa* genome has only one *tolC* homologue. According to Reddy and co-workers,^35^ a mutation of *tolC* in *X. fastidiosa* strain Temecula causes a severe loss of virulence in grape. In addition, in the same study, viable cells of *tolC* mutants were not recovered after inoculation into the grape xylem. This indicates that the efflux pump in which TolC is involved is critical to the survival of this pathogen within the xylem. Bacterial efflux is one of the primary systems responsible for the emergence of multidrug resistance, since efflux machineries can pump out several different types of antimicrobials.^36,37^ TolC homologues play a role in resistance against antimicrobial compounds in mammalian pathogens such as *Escherichia coli* or *Salmonella enterica*, as well as in plant pathogens like *Erwinia amylovora*.^38^ Therefore, targeting TolC implicates not only an impairment in *X. fastidiosa* virulence but also in potential resistance mechanisms of the bacterium.

XpsE and XpsG belong to the type II secretion system (T2SS). Ingel et al.^39^ demonstrated that *X. fastidiosa* strain Temecula, deficient of both proteins, is not pathogenic *in planta*, indicating a strong connection between the T2SS and the secretion of critical cell wall-degrading enzymes for plant colonization.

XatA is a protein belonging to the Type V secretion system, which was deeply characterized by Matsumoto et al.;^40^ according to this study, *xatA* mutant of *X. fastidiosa* Temecula1 is impaired in autoaggregation and in forming biofilm *in vitro* conditions. Moreover, when inoculated into grapevines, the mutant strain was compromised in plant migration and colonization, inducing few, if any, PD symptoms compared with the wild-type parent strain.

Taken together, these findings indicate that multiple secretion systems in *X. fastidiosa* are not redundant but functionally coordinated to ensure survival within the xylem environment and effective host colonization. The severe attenuation observed in mutants affecting type I, II, and V secretion systems highlights that extracellular protein export is a central virulence determinant. Importantly, because these systems mediate both pathogenicity and stress adaptation, they represent attractive intervention points, where their disruption could simultaneously impair virulence and bacterial persistence.

### 5.2 Hydrolytic Enzymes/Cell Wall Degrading Enzymes (CWDEs)

The role of the lipase/esterase LesA in *X. fastidiosa* pathogenicity has been highlighted by Nascimento and co-workers.^41^ In this study, LesA has been identified as the most abundant protein in the *X. fastidiosa* secretome. Grapevine inoculation with the *lesA* mutant showed much fewer PD symptoms compared to the plants inoculated with the unaltered strain.

Two *X. fastidiosa* β-1,4 endoglucanases (EG), EngXCA1 and EngXCA2, also affect bacterial virulence. EGs are hydrolytic enzymes that can digest xyloglucans by cleaving linkages in the glucan backbone, such as those found in cellulose and hemicellulose, two constituents of the vascular plant cell wall.^42,43^ *X. fastidiosa* mutants missing *engXCA1* and *engXCA2* genes in tandem resulted impaired in both virulence and population size.^44–46^

Among CWDEs, polygalacturonases (PG) are also important virulence factors;^47,48^ this class of enzymes catalyzes the hydrolytic cleavage of pectic polymers in plant cell walls. *X. fastidiosa* subsp. *fastidiosa* genome encodes for only one PG (PglA), which was characterized by Roper et al. These studies show that the *pglA* mutant of the PD strain *X. fastidiosa* subsp. *fastidiosa* Fetzer failed to move beyond the point of inoculation and did not induce PD symptoms, indicating that the PG is required for colonization and pathogenicity in grapevines.^46^

Labroussaa et al.^49^ characterized a functional Chitinase identified in the *X. fastidiosa* genome (ChiA), demonstrating that the *X. fastidiosa* subsp. *fastidiosa chiA* mutant strain was unable to grow on chitin as the sole carbon source. Moreover, mutants are limited in bacterial multiplication, transmission by vectors to plants, and plant colonization.

CWDE mutants consistently showed impaired plant colonization and reduced symptom development. As these enzymes act extracellularly or in the periplasm, they could offer high accessibility to small-molecule inhibitors.

### 5.3 Proteins Involved in Quorum Sensing

Extracellular protease expression in *X. fastidiosa* is among the traits controlled by the quorum-sensing mechanism; this is a general strategy that allows bacterial cells to sense and respond to changes in their populations through cell-to-cell communication.^50,51^ As occurs in many *Xanthomonas* species, in *X. fastidiosa*, quorum sensing is fine-regulated by a small molecule called diffusible signaling factor (DSF), which is a fatty acid derivative.^52,53^ DSF is synthesized by the protein RpfF.^54^ When DSF reaches a threshold concentration outside the cell, the RpfF-RpfC interaction is activated.

RpfF and RpfC were studied in *X. fastidiosa* by Newman et al., who created mutant strains where the *rpfF* gene was disrupted; they exhibited hypervirulence when needle-inoculated in grapevines but were deficient in colonizing insect vectors and, therefore, were not transmitted to new hosts.^54^ Mutants of *X. fastidiosa* blocked in the expression of *rpfC* exhibited a phenotype opposite to that of lacking *rpfF*: they overproduced DSF compared with the wild-type strain and showed reduced virulence and impairment in migration along the xylem vessels. *rpfC* mutants could be acquired by insects upon feeding on infected plants but were somewhat deficient in insect transmission.^55^

The gene *rpfB* also belongs to the *rpf* cluster, but in *X. fastidiosa*, it is located elsewhere in the chromosome, away from the *rpf* operon. Analysis of *X. fastidiosa* mutants missing *rpfB* showed a reduced level of insect colonization and transmission. Apparently, the RpfB protein is involved in DSF processing.^56^

The contrasting phenotypes observed in the rpfF and rpfC mutants underline the finely tuned regulatory balance required for optimal virulence. Hypervirulence *in planta* coupled with reduced vector transmission suggests that *X. fastidiosa* pathogenic success depends on a dynamic modulation between planktonic and biofilm lifestyles.

*X. fastidiosa* mutants lacking the *cgsA* gene analyzed by Chatterjee and co-workers showed a similar phenotype to the *rpfC* knockout strain, being hyperadhesive and weakly virulent to plants, while transmissible by insect vectors.^57^ The *cgsA* gene encodes a GGDEF domain protein likely involved in the synthesis of cyclic di-GMP.

### 5.4 Regulatory Systems

The regulatory gene *algU* intervenes in stress response and regulation of the biosynthesis of the exopolysaccharide alginate in *Pseudomonas aeruginosa* and other bacteria.^58^ *X. fastidiosa* mutants lacking the *algU* gene showed reduced biofilm formation, as well as impaired cell–cell aggregation and attachment, and lower virulence in grapevine.^59^

Less biofilm production and slow disease progression, compared to wild-type, were also observed in *pilL* mutants of *X. fastidiosa*. The *pilL* gene encodes a protein necessary for the bacterial twitching motility.^60^

The highly conserved two-component regulatory system PhoP/PhoQ has been reported to be essential for *X. fastidiosa* survival *in planta*.^61^ PhoQ is a transmembrane histidine protein kinase capable of detecting a range of stimuli, including acidic pH, antimicrobial peptides, Mg2+, and Ca2+. PhoP is a DNA-binding response regulator that, after phosphorylation, binds to its target DNA sequences and modifies transcription of the corresponding genes.^62^ According to the study of Pierce and co-workers, *X. fastidiosa* mutants showed no pathogenicity when inoculated in grapevine; therefore, no PD symptoms have been observed in infected plants.^61^

An additional two-component regulatory system widely studied in Gram-negative bacteria is the GacS/GacA system.^63^ Shi and co-workers^64^ showed that *X. fastidiosa*-Δ*gacA* had significantly reduced abilities of surface attachment, biofilm formation, and less severe disease symptoms in grapevines, compared with the wild type. A *gacS* gene homologue was not found, suggesting that there may be a specific regulatory role for *gacA* in *X. fastidiosa*.^15^

Voegel and co-workers characterized XhpT knockout mutants of *X. fastidiosa*, demonstrating that this protein is essential for overall virulence in grapevine, being implicated in the regulation of biofilm formation and exopolysaccharide (EPS) production.^65^

### 5.5 Biosynthesis of Extracellular Polysaccharides

Killiny and associates^66^ showed that disruption of two genes belonging to the gum operon of *X. fastidiosa* impacts EPS production. The genes *gumD* and *gumH* were knocked out in this study, and both mutants produced significantly less EPS compared to the wild type and were deficient in biofilm formation. Consequently, being impaired in movements within plants, mutant strains proved to be not pathogenic to grapevine and were very poorly transmitted by insect vectors.

Clifford and co-workers targeted a key O-antigen biosynthetic gene, encoding for the Wzy polymerase. In this study, the behavior of a mutant strain of *X. fastidiosa* lacking the *wzy* gene was analyzed.^67^ Significantly fewer PD symptoms were observed in grapevines inoculated with the *wzy* mutant compared to those of wild-type-inoculated plants. The mutant strain was impaired in cell–cell aggregation and biofilm maturation, two critical steps for successful infection of the host.

Bacterial cells arranged in a biofilm complex have some adaptation advantages, such as increased resistance to antimicrobial agents. In fact, the exopolysaccharide matrix represents an initial barrier that can delay penetration of drugs. Targeting genes involved in the biofilm formation process could exert reduced selective pressure, potentially limiting resistance emergence.^68,69^

### 5.6 Movement- and Attachment-Related Proteins

Artificial inoculations with *X. fastidiosa* subsp. *fastidiosa* strain Temecula mutants lacking both FimA and FimF proteins, which constitute, respectively, the major subunit and the anchor protein of the main fimbrial structure of short pili, proved that the virulence of the mutated strains was compromised and the bacterial populations in plants were slightly lower than those in grapevines inoculated with the wild-type strain.^70,71^ Killiny and Almeida^72^ demonstrated that the initial adhesion and maintenance of the bacterial biofilm in the insect foregut is also mediated by FimA, since the *X. fastidiosa* strain defective for the adhesin encoded by this gene was less efficiently acquired and retained by the vectors.

XadAs are membrane-associated proteins identified in the outer membrane vesicles and putatively assigned to the family of trimeric autotransporter adhesins; XadA1 has previously been shown to be involved in all steps of biofilm formation, whereas XadA2 has a role in the attachment to the surfaces,^73,74^ and XadA3 strongly contributes to cell–cell bacterial aggregation. *xadA1* and *xadA2* mutant strains exhibited reduced adhesiveness to glass surfaces and conferred a decreased disease severity in infected grapevines compared with those inoculated with the wild-type strain.^71^ Conversely, *xadA3* deletion causes a reduction of biofilm formation, due to the deficiency in cell–cell aggregation of the mutant and promotes a hypervirulent phenotype in plants; however, Δ*xadA3* greatly impairs the vector transmission of *X. fastidiosa* cells.^75^

MopB is a major conserved outer membrane protein of *X. fastidiosa* subsp. *fastidiosa*. As demonstrated in a previous study,^76^ *mopB* deletion causes loss of twitching motility by hindering the biogenesis of type I and IV pili and undermining the membrane integrity. As confirmation, tobacco plants inoculated with the Δ*mopB* strain exhibited slow symptom development and reduced disease severity. Moreover, MopB has been utilized as a molecular target in an antimicrobial treatment strategy against *X. fastidiosa* that employs a synthetically designed protein chimera.^77^

In Gram-negative bacteria, the TonB system is involved in iron and vitamin B12 transport. Cursino and co-workers^78^ showed that a mutant of a *tonB* homologue, named *tonB1*, in the Temecula1 strain, was affected in twitching motility and biofilm formation, producing colonies with a smooth periphery and a thinner biofilm layer. In addition, the severity of disease symptoms in plants inoculated with the Δ*tonB1* was significantly less than in grapevines inoculated with the wild-type strain.

### 5.7 Toxins

The *zot* gene (*Zonula occludens* toxin) in *X. fastidiosa* encodes a bacterial exotoxin, most likely belonging to the *Inoviridae* family.^79,80^ Although this gene is annotated in multiple copies in several *X. fastidiosa* genomes (Temecula1, M23, GB 514, Ann-1, etc.), its function and mechanism of action remain poorly investigated. By inoculating grapevines with a transformant EB92-1 strain expressing the *zot* genes, Zhang and co-workers^81^ demonstrated that Zot-like proteins are necessary for pathogenicity, obtaining visible symptoms on leaves starting from 6 weeks after inoculation.

The CvaC/Cvi couple represents a toxin-antitoxin (TA) system; the bacteriocin encoded by the *cvaC-1* gene, the top-regulated and stable transcript produced by *X. fastidiosa* subsp. *pauca* ST53 during the infection process in olive,^82^ is a pore-forming toxin that directly damages the plasma cell membrane of competitive endophytic bacteria present in the xylem vessels and insect foregut,^83^ while concurrently, *X. fastidiosa* also expresses the immunity protein Cvi to protect itself from the harmful effects of its own antibacterial product.^84^ Such a TA system may not only contribute to niche advantage over other taxonomically related bacterial species inhabiting the xylem environment but could also indirectly influence bacterial virulence and persistence within the plant host. Indeed, CvaC possibly plays a structural role in biofilm maturation.^85^ Knockout mutants of *cvaC-1* and *cvi* genes of Temecula1 and De Donno strains showed a significant reduction in growth and biofilm formation compared to the corresponding wild-type cultures (Amoia et al., unpublished data), thus confirming that both genes are also involved in bacterial survival. Moreover, the *cvi* deletion significantly slows down the formation of fringes in the Temecula mutant strain (Amoia et al. unpublished data). Targeting TA systems would also affect the antimicrobial compound tolerance. In fact, a transcriptional profile study of *X. fastidiosa* subsp. *pauca* in response to copper and tetracycline showed the induction of TA systems, associated with the formation of persister cells.^68^

### 5.8 Stress Response

Cold shock proteins (Csps) control virulence-associated genes by acting as nucleic acid-binding chaperones and thereby contribute to bacterial pathogenicity.^86^ A knockout mutant c*sp1* in *X. fastidiosa* subsp. *fastidiosa* (Δ*csp1*) showed reduced stationary-phase survival, biofilm formation, and surface adhesion with limited production of pili.^87,88^ PD-susceptible Chardonnay grapevines inoculated with Δ*csp1* produced notably reduced symptom severity and harbored a lower bacterial titer without any cold stress event, making this protein temperature-independent.^89^

### 5.9 Secondary Metabolism

Fatty acyl-CoA synthetase (ACS) likely plays a key role as an intermediate enzyme in several metabolic pathways such as fatty acid production, membrane composition and integrity, and energy production. Lindow and co-workers^90,91^ proved that the putative ACS, encoded by the PD1311 gene and highly conserved across *X. fastidiosa* subspecies, is a decisive virulence factor involved in the establishment of the infection. The authors carried out both *in vitro* and *in planta* assays by comparing the phenotype of the PD1311 deletion mutant (ΔPD1311) and the corresponding wild-type. After 24 weeks post inoculation, susceptible grapevine cultivar Cabernet Franc inoculated with ΔPD1311 showed a statistically significant reduction of PD severity compared to those inoculated with the wild-type.

### 5.10 Practical Relevance of Virulence Factors as Targets for Crop Protection

Overall, the genetic and functional studies summarized above demonstrate that *X. fastidiosa* virulence relies on a tightly interconnected network of secretion systems, extracellular enzymes, regulatory pathways, biofilm determinants, and stress-response mechanisms. Targets whose disruption completely abolishes xylem colonization (such as CWDEs, T2SS components, TolC, and PhoP/PhoQ) are considered high-value candidates, because their inhibition is expected to impair essential steps of infection even without bacterial eradication. By contrast, targets involved in quorum sensing or surface adhesion often reduce but do not eliminate virulence and therefore may provide partial protection or require combination approaches. In practical application, the actual degree of protection will depend on factors such as compound stability, uptake, and delivery into xylem vessels and the extent to which chemical inhibition mimics the genetic knockout. While these aspects remain to be investigated experimentally for *X. fastidiosa*, the phenotypic profiles of the validated virulence factors provide a rational basis for prioritizing targets for structure-based inhibitor development.

## 6 Target Assessment: Structural Knowledge, Protein Production, Crystallization, and Ligand-Binding-Site Predictions

### 6.1 Structural Knowledge on Virulence Factors

To date, none of the proteins listed in Table 1 has been structurally characterized. Some proteins from *X. fastidiosa* have been studied by X-ray diffraction (Supporting Information, Section S1) but there are no known virulence factors among them. A rational structure-based drug discovery approach requires atomic-resolution three-dimensional structures of target proteins, and X-ray diffraction (XRD) remains the main technique for experimental structure determination. It involves a series of experimental steps, such as protein production and purification, crystallization, and X-ray data collection, followed by computational steps including indexing, initial phasing, phase refinement, model building, its refinement, and validation. While the computational procedures are implemented in well-established computer programs,^92–95^ with generally positive outcomes if the data quality is good, experimental steps, particularly crystallization, still pose significant challenges. Therefore, to prioritize protein targets from Table 1 amenable to structural studies, their crystallizability was predicted using the XtalPred server.^96^ This tool evaluates biochemical and biophysical features derived from the protein sequence and assigns a crystallization probability class via a Random Forest classifier, by choosing among 11 classes, ranging from optimal (1) to very difficult (11) crystallization. In the case of proteins composed of different functional domains, the crystallizability of individual domains could be higher than that of the full-length protein. For proteins with poor crystallization predictions, XtalPred also suggests optimized constructs to improve crystallization success without compromising protein functionality. When crystallization remains unfeasible, alternative methods such as cryo-electron microscopy (Cryo-EM), particularly suited for large macromolecules (>100 kDa), and nuclear magnetic resonance (NMR), appropriate for smaller proteins (<25 kDa), can be applied.

Regardless of the methods, the successful recombinant expression of soluble proteins remains essential. Therefore, a selection criterion based on predicted soluble expression, assessed through the machine learning performed on the SoluProt server,^97^ was applied jointly with the one based on crystallization prediction. Therefore, selected targets reported in Table 2 correspond to those with the highest predicted crystallization propensity, for the whole protein or for individual functional domains, and/or with favorable soluble expression predictions.

**Table 2:**
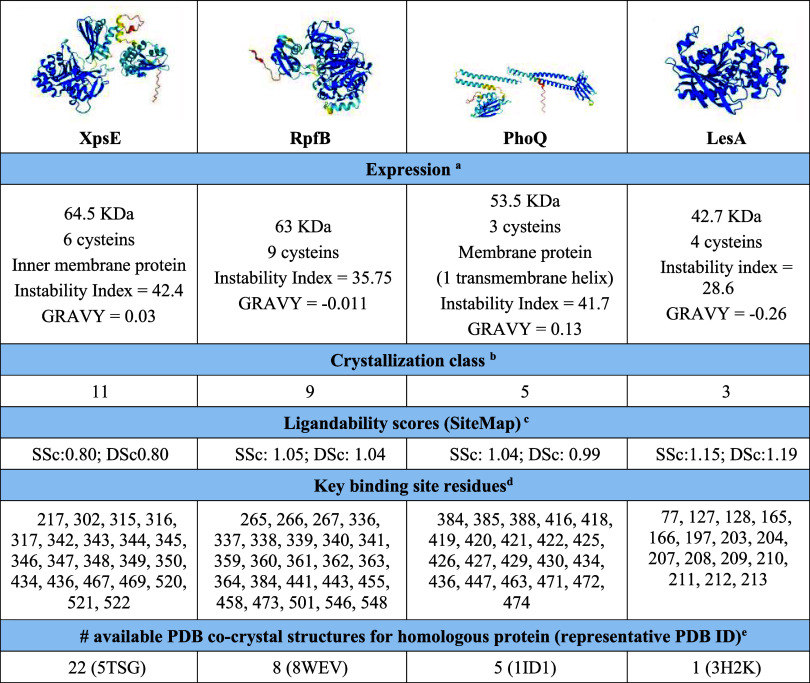
Proposed *X. fastidiosa* Targets for Rational Drug Design of Agrochemicals

a The instability index is used to determine whether a protein will be stable in a test tube. If the index is less than 40, then it is probably stable in the test tube. If it is greater than this, it is probably not stable. The grand average of hydropathicity index (GRAVY) is used to represent the hydrophobicity value of a peptide, which calculates the sum of the hydropathy values of all the amino acids divided by the sequence length. The GRAVY value was calculated using the hydropathy values from Kyte and Doolittle. Positive GRAVY values indicate hydrophobicity; negative values mean hydrophilicity.

b The crystallization class, predicted by a random forest classifier, ranges between 1 (most promising) and 11 (least promising). The prediction refers to the entire protein and not to their individual domains.

c SiteScore (SSc) and DrugScore (DSc) values ≥ 0.8 distinguish between drug-binding and nondrug-binding sites.^114^

d Key residues represent the overlap between SiteMap-predicted pocket residues and ligand-binding residues (within 5 Å of cocrystallized ligands) in homologous proteins; ^d^Reported residues belong to domains that have at least a homologue with known crystallographic structure at RMSD < 2 Å;

e Number of cocrystal structures sharing ≥40% sequence identity with the target; in parentheses, the PDB ID of the protein with the highest sequence identity, selected as representative structure. For a complete list of PDBs, refer to Table S1 in Supporting Information.

In the absence of experimentally determined structures, computational approaches based on homology modeling and artificial intelligence (AI) offer powerful alternatives. The extensive data set of Protein Data Bank (PDB)^98^ provides structural templates that enable accurate predictions using trained neural networks. Currently, AlphaFold^99^ represents the state-of-the-art tool, generating complete models accompanied by a residue-by-residue score (pLDDT) that indicates the confidence of the prediction on a scale from 0 to 100. As such, predicted structures can be used as a valid alternative to those experimentally determined.

Overall, the selection of targets reported in Table 2 combines the evaluation of their biological relevance in *X. fastidiosa* virulence and their structural suitability for SBDD. The four chosen targets represent the subset of virulence determinants that simultaneously offer antivirulence potential and practical feasibility for structure-guided inhibitor discovery.

In order to analyze target conservation between different *X. fastidiosa* subspecies, we performed multiple sequence alignments for each one of the four selected targets (Figure S2). The alignments include representatives of the five main *X. fastidiosa* subspecies *fastidiosa*, *pauca*, *multiplex*, *sandyi*, and *morus*. Three of the targets, XpsE, RpfB, and PhoQ, show a very high level of conservation, with sequence identities of 99.4%, 98.5%, and 99.3%, respectively, across all subspecies. LesA sequences show less identity (87,8%), reflecting subspecies-specific divergence. The reduced conservation in some of the predicted key binding sites residues suggests that agrochemicals targeting LesA will require subspecies-tailored optimization, and consequently, customization according to the associated plant disease.

#### 6.1.1 Validation of AlphaFold Structures for Selected Virulence Factors

The reliability of the structural models generated by *in silico* algorithms can be assessed by comparing the predicted folding of individual domains to that of homologous proteins with experimentally determined structures, in order to achieve a more direct measure of structural accuracy than AlphaFold’s internal confidence score (pLDDT). The steps needed to perform such validation are the following: search for homologues with known structure from protein sequence, identification of protein domains from structural models, structural alignment of individual domains, and calculation of residue-by-residue distance metrics from aligned structures. We applied this pipeline to the AlphaFold models of selected protein targets for *X. fastidiosa* listed in Table 1, using tools described in Supporting Information (Section S2). Examples of results of the validation process are shown in Figure S1. Only residues of AlphaFold structures included in regions showing RMSD < 2 Å were considered reliable for druggability predictions, while others were interpreted with caution.

### 6.2 Ligandability Analysis of Virulence Factors

A central advantage of having access to a 3D structure of a protein target is the possibility to directly investigate its ability to host small molecules within specific cavities on the protein surface. Not all pockets, however, are equally suitable for ligand accommodation, and the protein “ligandability” (*i.e*., the ability of a protein target to bind small molecules with high affinity) depends on the presence of cavities having favorable geometric and physicochemical features.^100^ In general, ligandable pockets display an appropriate volume, typically in the range of 160–800 Å^3^, which is large enough to accommodate small molecules but not so extended as to lose specificity.^101^ They also exhibit a favorable distribution of hydrophobic and polar regions, since hydrophobic interactions are among the main driving forces of ligand binding, while selective hydrogen bonds often fine-tune affinity and orientation.^102,103^ In addition, the extent of pocket enclosure and depth strongly influences binding strength, as more buried cavities reduce solvent competition and provide a higher number of complementary contacts with ligands.^104^ These properties, taken together, define the structural determinants that enable a cavity to act as an effective binding site.

In this context, several computational approaches have been developed to identify and characterize potential binding sites directly from protein structures,^105,106^ including tools such as SiteMap,^107,108^ DoGSiteScorer,^109^ Fpocket,^110^ and DeepSite.^111^ More recently, the integration of AI-based and deep learning methods has further expanded pocket detection capabilities, exemplified by tools such as InDeepNet^112^ and LABind.^113^

Valuable information can also be obtained by structural alignment with homologous proteins at known binding sites. Conserved binding motifs or cocrystallized ligands in related structures provide valuable support for the presence of analogous pockets in the predicted model, anchoring computational predictions to empirical evidence. This integration of unbiased pocket detection with comparative structural analysis is particularly useful in target prioritization workflows, where identifying the most promising binding sites is essential for downstream docking and virtual screening campaigns.

Following the above-described principles, we generated a prioritized list of putative binding pockets for each protein in Table 1 (see example in Table S1) by using SiteMap^114^ as a pocket scouting tool, a list that was further refined by considering three complementary criteria:

(1) Availability of structural data from homologous proteins—presence of cocrystallized ligands in homologues strengthens the plausibility of analogous binding sites in our target.
(2) Biological accessibility of the predicted pockets.
(3) Structural reliability of the pocket region in the AlphaFold model—pockets located in low-confidence or disordered regions were deprioritized (see Supporting Information, Section S2).

By integrating this information, we generated a refined short list of promising binding sites (shown in Table 2).

As an illustrative case study, Figure 3 reports the results obtained from the ligandability analysis of the virulence factor LesA. The SiteMap investigation revealed the presence of a favorable pocket characterized by promising scores (SiteScore: 1.154; DrugScore: 1.191). In parallel, a search in the PDB identified a homologous esterase from *Xanthomonas oryzae* ^115^ (LipA; PDB ID: 3H2K), sharing a high 67.7% sequence identity with LesA. The LipA structure was cocrystallized with a β-octylglucoside (BOG) molecule, which has been reported to mimic its natural substrates. BOG binds to a site corresponding to that identified by the SiteMap analysis on LesA, thus supporting the relevance and reliability of the predicted binding pocket.

**Figure 3:**
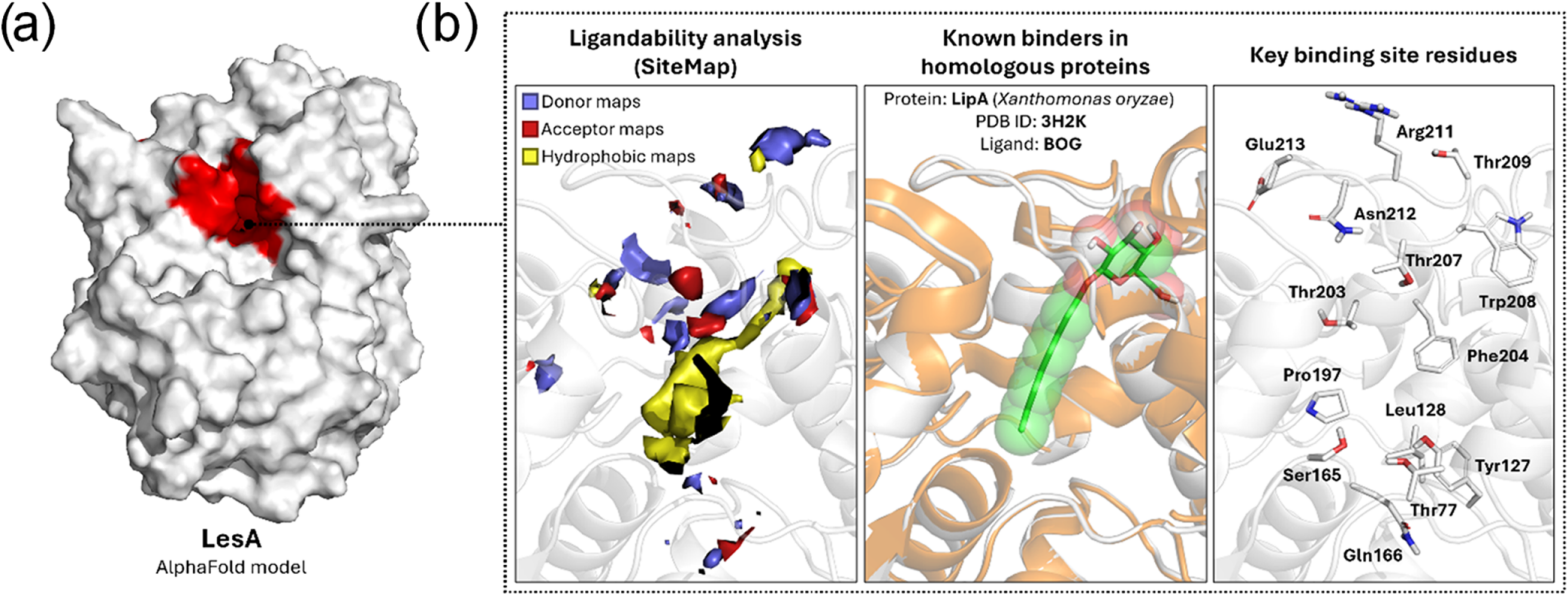
Results of ligandability analysis for LesA from *X. fastidiosa* subsp. *fastidiosa*. The LesA active site (red region in a) is structurally analyzed in terms of residue properties, interaction with ligands, such as BOG: octyl β-d-glucopyranoside, and residues involved in active-site interactions (b).

Overall, this analysis illustrates a practical and reproducible framework not only for binding-site identification but also for the prioritization of AlphaFold-derived targets in view of SBDD (paragraph 7). More specifically, the proposed short list of targets and associated pockets is intended to highlight those systems for which, based on the currently available evidence (*i.e*., presence of homologous ligand-bound structures) and preliminary *in silico* pocket scouting analyses, the likelihood of identifying a plausible and ligandable site is higher. This type of assessment is essential for selecting the protein targets that provide the most promising starting points for a discovery project with a high potential for success. At the same time, these results should be regarded only as an initial step within a broader validation workflow, in which the proposed binding site must ultimately be supported by experimental evidence, such as X-ray crystallography or mutagenesis studies, to confirm its structural plausibility, effective ligandability, and relevance for protein modulation. In parallel, AlphaFold-derived models may benefit from further computational refinement, including molecular dynamics simulations, to explore the conformational landscape of the selected pocket and identify the most suitable conformations for the downstream discovery strategies discussed in the next section.

## 7 *In Silico* Identification of Potential Agrochemicals

Starting from the 3D structures of the selected *X. fastidiosa* targets (Table 2), different computational approaches can be applied within a rational, data-driven workflow to identify hit compounds, *i.e*., chemical entities capable of binding to and modulating the target protein with acceptable activity in primary assays, providing evidence of target engagement and serving as tractable chemical starting points for subsequent steps. In particular, this section focuses on two of the most widely adopted strategies in the human drug discovery field: structure-based virtual screening (SBVS) and fragment-based drug discovery (FBDD). SBVS^24–26^ systematically docks large, prefiltered libraries of small molecules into a protein binding site to predict binding modes and relative affinities, enabling rapid prioritization of candidates for experimental testing. Docking engines commonly used for this purpose include Glide (Schrödinger),^116^ AutoDock Vina,^117^ Gold (CCDC),^118^ MOE (Molecular Operating Environment),^119^ Dock,^120^ and BioSolveIT.^121^

Screening libraries may be derived from commercial vendors (*e.g*., Enamine, ChemBridge, or MolPort) or in-house collections.

As in human drug discovery, the choice of library and starting scaffolds is not pathogen-specific *per se*, but depends on the target class, intended application route, and project constraints (*e.g*., assay format, synthetic capability, availability of in-house chemistry, and intellectual property considerations). At the same time, optimal enrichment with natural product-derived or biodegradable chemotypes can be considered, as these structures may combine biological relevance with favorable environmental characteristics.^122^ Docking results are rescored and refined using consensus schemes and physics-based methods such as Molecular Mechanics/Generalized Born Surface Area, providing more reliable energy-based rankings to improve hit quality.^123,124^

Increasingly, AI-based models are being integrated into SBVS workflows to further enhance ranking accuracy and reduce false positives.^125–127^

The final output of SBVS is a short list of “virtual hit” compounds, each supported by a plausible binding hypothesis, ready for experimental confirmation. At this stage, simple *in silico* property filters help prioritize assay-suitable, chemically developable compounds, focusing in particular on aqueous solubility and moderate lipophilicity (LogP/LogD) to minimize aggregation, nonspecific effects, and early false positives. FBDD^128–130^ offers a complementary or alternative strategy to identify virtual hits from predicted 3D structures of molecular targets. Instead of docking full-size molecules as in SBVS, small fragments (usually <300 Da and selected from specialized fragment libraries) are computationally screened to detect weak yet highly efficient binders that reveal interaction hot spots within the pocket. Fragment libraries may derive from commercial collections (*e.g*., Astex, Maybridge, or BioSolveIT’s FragSpace) or curated in-house sets optimized for diversity and synthetic tractability. Because of their small size and low affinity, fragment “virtual hits” cannot typically elicit measurable biological effects in cellular or organismal assays; therefore, they must first be experimentally validated on the purified target, using techniques such as NMR, Surface Plasmon Resonance (SPR), or XRD, and subsequently elaborated through fragment-growing, linking, or merging strategies to generate higher-affinity molecules suitable for downstream biological testing.^131,132^ Regardless of the approach used (SBVS or FBDD), only candidates confirmed through multiple orthogonal assays (Paragraph 8) progress into the Hit-To-Lead (H2L) and Lead Optimization (LO) phases. These stages refine potency, selectivity, and baseline developability through iterative design-make-test-analyze (DMTA) cycles guided by emerging Structure–Activity Relationships (SAR) and, when available, high-resolution structural data (*e.g*., X-ray cocrystals or cryo-EM reconstructions).

Critically, H2L and LO are where the program transitions from potency-driven refinement to true multiparameter optimization under agro-specific constraints. Alongside improving target potency against the selected bacterial virulence targets, campaigns should use iterative structure–property optimization to steer compounds toward profiles compatible with *in planta* performance, most notably systemic uptake and xylem mobility. Importantly, as in pharmaceuticals (*e.g*., oral vs topical delivery), physicochemical requirements depend on the intended application route (*e.g*., foliar, soil, or trunk injection/endotherapy), and thus xylem exposure should be optimized accordingly using key descriptors such as LogD/LogP at relevant pH, aqueous solubility, p*K*_a_/ionization, and molecular size (*e.g*., molecular weight, MW). This property tuning should be addressed early, as formulation can support delivery but cannot fully compensate for intrinsically unsuitable physicochemical profiles. In parallel, sustainability and safety can be addressed by early *in silico* derisking of environmental fate and ecotoxicity liabilities, including predicted persistence/biodegradability, bioaccumulation potential (*e.g*., bioconcentration factor, BCF), and potential impacts on representative nontarget organisms relevant to agricultural ecosystems (*e.g*., pollinators and other beneficial insects, natural predators, soil microbiota and invertebrates, aquatic species, birds, and wild mammals), while ensuring adequate safety margins for operators and consumers under realistic use scenarios.^133–136^

In both cases, computational estimation can be supported by widely used QSAR/QSPR resources applied in regulatory and screening contexts (*e.g*., ECOSAR, EPI Suite, OPERA, and the OECD QSAR Toolbox) as well as newer multiend point AI platforms (*e.g*., Deep-PK)^137^ for rapid parallel profiling and prioritization, provided their applicability domain is considered.

## 8 Experimental Validation: Multilevel Biological Assays for *In* *Vitro* and *In Planta* Studies on Candidate Agrochemicals

The discovery and development of new agrochemicals require a rigorous validation process supported by orthogonal assays at multiple levels of complexity. These assays are not confined to a single stage but rather represent a recurring necessity throughout the entire pipeline, from hit identification, through H2L optimization, up to the final selection of lead candidates. At each step, they provide complementary information: from confirming direct interaction with the molecular target to demonstrating functional activity in cellular and living systems and ultimately assessing efficacy and safety under agronomically relevant conditions.

The validation of potential hit compounds typically begins with *in vitro* assays using the isolated target protein. Depending on the nature of the target, different strategies can be employed: biophysical techniques, such as SPR or Isothermal Titration Calorimetry (ITC), directly measure binding affinity and kinetics, while biochemical assays are used to evaluate the compound’s ability to modulate target activity, such as inhibition assays for enzymatic proteins.

Validation at the isolated protein level, however, is not sufficient to fully establish biological relevance. For this reason, cell-based assays are required to determine whether the compound can modulate the target within the complexity of a biological system while accounting for factors such as cell permeability, target accessibility, and potential off-target effects.

As in human drug development, where clinical trials are necessary to confirm efficacy and safety in patients, the agrochemical pipeline requires *in planta* assays under controlled conditions to verify the compound’s effectiveness against the target organism. For xylem-limited pathogens such as *X. fastidiosa*, validation at this stage should also account for exposure at the site of infection, *i.e*., whether the compound can achieve sufficient systemic uptake and xylem mobility to reach the bacterial niche. These are followed by field trials, which are essential to evaluate real-world performance, environmental impact, and long-term sustainability before regulatory approval and eventual commercialization.

At the conclusion of this iterative, multilevel validation process, a candidate agrochemical is selected based on a balanced profile of efficacy, *in planta* exposure (xylem delivery), and environmental safety, supported by an appropriate physicochemical property space. In the context of *X. fastidiosa*, multilevel validation strategies can be implemented through a wide range of antibacterial assays *in vitro* and *in planta*, as summarized in Table 3 together with their purpose, targeted subspecies, representative compounds tested, and key references.

**Table 3:** *In Vitro* and *In Planta* Assays Used to Test Potential Agrochemicals against *X. fastidiosa*

	assay	purpose/description	subspecies	compounds	references
*In vitro* antibacterial assay	*In vitro* planktonic growth inhibition	Measures bacterial planktonic growth in liquid media in the presence of antimicrobial compounds. Serial dilutions are performed to identify the Minimum Inhibitory Concentration (MIC) or Minimum Bactericidal Concentration (MBC)	*fastidiosa, multiplex, pauca*	Coumarin, catechol, naringenin, quercetin, rutin, catechin, resveratrol, thymol-nanoparticle, Antimicrobial Peptides (AMP), Olive Pomace Phenolics (OPP) extract, melittin, Scorpine-Like Molecule (SLM), gomesin	^138–144^
	PCR-based bacterial quantification; viability −qPCR (v-qPCR)	Quantifies viable bacterial populations post-treatment by discriminating between live and dead cells. Often used to confirm the efficacy of the molecule against *X. fastidiosa* *in vitro* or *in planta*	*pauca*	*N*-Acetylcysteine, AMP (1036, RIJK2, BP100), nisin	^145–149^
	Biofilm inhibition	Evaluates inhibition of biofilm formation on abiotic surfaces.	*pauca, multiplex, fastidiosa*	Micronutrients, metal salts, phenolic compounds, antibiotics, Dentamet, gomesin	^138,143,146,150^
	Fluorescent Live/Dead Cell Viability Assay	Differentiates live/dead bacteria post-treatment using fluorescent dyes. A visual method to monitor the bacterial population alive.	*pauca*	Thymol-nanoparticle,	^144^
	Microfluidic-Based Antimicrobial Susceptibility Testing	Method based on microfluidic channels. Evaluates the effect on the molecules mimicking the behavior of *X. fastidiosa* growing in xylem vessels.	*fastidiosa*	Copper sulfate, *Punica granatum* extract, Trametes versicolor extract, Clove oil, Fossil	^151^
*In vitro* antibacterial assay	Time-kill contact assay	A complementary assay to MIC and MBC to evaluate the potential inhibition of cell growth.	*pauca*	Metal ions, micronutrients, antibiotics, and phenolic compounds, OPP extract	^138,141,152^
	Agar diffusion assay	Method based on the diffusion of antimicrobial agents in agar medium, where bacteria are inoculated. The sensitivity of bacteria to the agent is evaluated based on the resulting growth inhibition area	*fastidiosa, multiplex, sandyi pauca*	Metal ions, micronutrients, antibiotics, phenolic compounds, AMP, OPP extract, olive mill wastewaters, fungal extracts, toxins, flavonoids, coumarins, alkaloids, dihydrocinnamic acid derivative, anacardic acid, triterpenes, limonoids	^138,140,141,148,152–155^
	Agar dilution assay				
	Antibiogram assay				
	Transcriptomic analysis/Gene expression	to evaluate the expression of genes associated with bacterial growth, biofilm, multidrug resistance, and other functions.	*pauca*	Copper sulfate	^156,157^
	TEM and optical microscopy	To evaluate cell morphology and phenotype with high-quality resolution by using optical or Transmission Electron Microscopy (TEM)	*pauca, fastidiosa*	Copper sulfate, *P. granatum* extract, Trametes versicolor extract, Clove oil, FossilⓇ, AMP	^148,149,151,157^
	Spot assay, turbidity reduction assay,	Mixtures (antibacterial + bacteria) are spotted onto the surface of the agar plates to see the sensitivity of the bacteria to the antibacterial.	*pauca*	Nisin	^149^
*In planta*	*In planta* colonization/antagonist assay	Monitors systemic colonization in plants post-treatment via qPCR or plating.	*pauca*	*N*-Acetylcysteine, nisin, gomesin.	^143,145,149^
	Field efficacy trials	Large-scale application of antimicrobials in natural environments for monitoring infection. The idea is to verify the efficacy of the substance in real conditions.	*pauca*	Phenolic extract, NuovOlivo, Dentamet	^158–161^
	Greenhouse *in vivo* biocontrol assays and bacterial population assessed by qPCR	To evaluate the effect of antimicrobial substances in controlled conditions (greenhouse) by monitoring *X. fastidiosa* population and symptoms after product application.	*pauca*	Tannins, fertilizers, micronutrients, and ions.	^152^
	Ionomic analysis	To evaluate alterations of leaves’ ionomic composition by using Inductively Coupled Plasma Atomic Emission Spectroscopy (ICP-AES).	*pauca*	Dentamet	^162^

Among the assays listed in Table 3, planktonic growth and biofilm inhibition assays, as well as time kill-contact, agar diffusion, spot or turbidity assays, dilution and antibiogram assays can be executed in medium/or high-throughput formats as described by Del Grosso et al.^138^ and therefore constitute the primary screening tier. Assays based on qPCR quantification (like viable qPCR), live/dead cell imaging, transcriptomic analysis, and TEM/optical microscopy operate at medium throughput and are used only for a reduced number of compounds selected from the initial screen. Tests that mimic the xylematic environment, like microfluidic chambers, and all *in planta* assays are intrinsically low throughput due to biological constraints and experimental duration. For this reason, compounds are gated between stages based on cumulative performance: only molecules showing consistent biochemical activity, reproducible antibacterial effects *in vitro*, and favorable physicochemical properties progress to the next level of validation.

The sustainable control of bacterial plant diseases remains a major challenge in modern agriculture and typically requires integrated management approaches rather than reliance on a single control measure. Ongoing strategies used to manage the persistent threat posed by *X. fastidiosa* can be summarized into four main approaches: eradication of infected plants, research of resistant germplasm, evaluation of compounds with antimicrobial properties, and control of insect vectors.^8^ In the framework of integrated pest management (IPM), the development of novel antibacterial molecules with high specificity, reduced environmental impact, and innovative mechanisms of action becomes essential. In this study, we propose a structure-guided drug discovery roadmap that outlines an integrated strategy for discovering antivirulence agrochemicals against this pathogen. By linking comparative genomics and functional genetics evidence compiled from the literature with structural/ligandability analyses, SBVS/FBDD, and tiered validation (*in vitro* and *in planta*), this strategy balances conceptual guidance with actionable steps. This roadmap offers a promising opportunity to design novel agrochemicals capable of selectively interfering with key bacterial processes while minimizing nontarget effects and resistance pressure. The aim is to ultimately strengthen IPM programs by shifting from broad-spectrum suppression to mechanism-informed and sustainable disease management.

Open data sharing between computational and experimental teams, as well as a cross-disciplinary integration among microbiology, molecular and structural biology, medicinal chemistry, and plant sciences, is critical to translating this roadmap into tangible outcomes: novel, safer, resistance-resilient, and environmentally responsible agrochemicals targeting *X. fastidiosa* that achieve enough specificity and selectivity, robust activity at practicable field doses with minimal effects on beneficial organisms, environmental compartments, and human health.

## Supplementary Material



## Notes

Additional data on *X. fastidiosa* known protein structures solved by X-ray diffraction and validation of predicted protein structural models (PDF)

## Abbreviations

PD: Pierce’s Disease
OQDS: Olive Quick Decline Syndrome
CVC: Citrus Variegated Chlorosis
IPM: integrated pest management
SBDD: Structure-Based Drug Discovery
NMR: Nuclear Magnetic Resonance
AI: Artificial Intelligence
CWDEs: Cell wall Degrading Enzymes
EG: Endoglucanases
PG: Polygalacturonases
DSF: Diffusible Signaling Factor
EPS: Exopolysaccharide
TA: Toxin-Antitoxin
ACS: Acyl-CoA Synthetase
XRD: X-ray diffraction
PDB: Protein Data Bank
RMSD: Root Mean Square Deviation
BOG: β-Octylglucoside
SBVS: Structure-Based Virtual Screening
FBDD: Fragment-Based Drug Discovery
H2L: Hit-to-Lead
LO: Lead Optimization
SAR: Structure–Activity Relationships
SPR: Surface Plasmon Resonance
IC: Isothermal Titration Calorimetry
MIC: Minimum Inhibitory Concentration
MBC: Minimum Bactericidal Concentration
TEM: Transmission Electron Microscopy
ICP-AES: Inductively Coupled Plasma Atomic Emission Spectroscopy
