# Supplementary material for: From Genome Inspection to Precision Agrochemicals: A Structure-Based Antivirulence Roadmap for Sustainable Crop Protection against Xylella fastidiosa
Source: J Agric Food Chem. 2026 May 26;74(22):16769–85. doi: 10.1021/acs.jafc.5c16967 (PMC13266967; doi:10.1021/acs.jafc.5c16967)
Supplement: Supplementary file 1 [file jf5c16967_si_001.pdf]

SUPPORTING INFORMATION

From Genome Inspection to Precision Agrochemicals: A Structure-Based Anti-Virulence Roadmap for Sustainable Crop Protection Against *Xylella fastidiosa*

Rosanna Caliandro [a], *Andrea Astolfi* [b], *Serafina Serena Amoia* [a], *Benny Danilo Belviso* [c], *Vincenzo Mangini* [c], *Reinaldo Rodrigues De Souza Neto* [a], *Anna Giovanna Sciancalepore* [c], *Stefano Sabatini* [b], *Annalisa Giampetruzzi* [a]*, *Maria Letizia Barreca* [b]*, *Rocco Caliandro* [c]*

[a] Institute for Sustainable Plant Protection, National Research Council (IPSP-CNR), via Amendola 122/D, 70126 Bari, Italy

[b] Department of Pharmaceutical Sciences, University of Perugia, Via del Liceo 1, 06126 Perugia, Italy

[c] Institute of Crystallography, National Research Council (IC-CNR), via Amendola 122/o, 70126, Bari, Italy

* Corresponding authors

Email: annalisa.giampetruzzi@cnr.it, maria.barreca@unipg.it, rocco.caliandro@cnr.it

S1 Experimental structures of *X. fastidiosa* proteins

The PDB includes experimentally resolved structures of *X. fastidiosa* proteins that, while not virulence factors or directly involved in pathogenicity, play important roles in cellular metabolism, stress response, and gene regulation. These structures are potentially valuable for expanding our understanding of bacterial biology and for identifying alternative targets in the design of precision agrochemicals. The proteins span a wide range of functional classes, including isomerases (e.g., PDB: 3FK8, 3GT5), chaperones (PDB: 5J7N), oxidoreductases (e.g., PDB: 1ZB8, 3IXR, 2REM), hydrolases (e.g., PDB: 5KSQ, 3OIS), transferases (e.g., PDB: 4NEX, 4NF1), transcription regulators (e.g., PDB: 3PQJ, 3PQK), and several proteins of unknown function (PDB: 2NUH, 2K5R).

For instance, the structure of the stationary phase survival protein E (SurE) (PDB: 5KSQ), a hydrolase involved in nucleotide metabolism, was resolved at 2.63 Å and provides insights into different oligomeric states (*e.g.*, dimeric vs. tetrameric forms), which may influence enzymatic activity. Similarly, a small heat-shock protein belonging to the chaperone proteins from *X. fastidiosa* 9a5c (PDB: 5J7N) may play a role in protein folding under stress conditions, potentially relevant during plant colonization. Transcriptional regulators like BigR (PDB: 3PQJ and 3PQK, representing reduced and oxidized states, respectively) suggest redox-sensitive regulatory mechanisms that may indirectly influence virulence or adaptation to the plant environment.

These structures, often solved at high resolution and expressed in *E. coli*, provide detailed views of active sites, cofactor interactions (*e.g.*, presence or absence of Mg^{2+} and Mn^{2+} in toxins such as PDB: 2OAI and 2R8D), and conformational changes upon ligand binding (as observed in ATP-bound vs. ATP-free forms of a transferase, PDB: 3LKI vs. 3LJS). These non-virulence-related protein structures enrich the structural biology landscape of *X. fastidiosa*, offering potential insights into druggability and highlighting complementary pathways for the development of novel antimicrobial strategies targeting essential physiological processes beyond traditional virulence factors.

Table S1. List of co-crystal structures sharing ≥40% sequence identity with proposed *X. fastidiosa* targets for rational drug design of agrochemicals.

Xf target	PDB ID (co-crystallized ligand)
**XpsE**	5TSG (ADP);5TSH (ANP, ADP);1P9W (ANP);4PHT (ANP);5OIU (ATP);5IT5 (ATP, AGS);8DZE (ANP);8DZF (ANP);8DZG (ADP);2EWV (ADP);2EWW (ATP);2GSZ (ADP);3JVV (ACP);5FL3 (ADP);6OJX (ATP);6OJZ (ADP);6OK2 (ADP);6OKV (ANP, ADP);6OLM (ATP);2OAP (ANP);8RJF (ADP);8RKD (ANP, ADP)
**RpfB**	8WEV (AMP); 6K4C (SLU); 6K4D (D4F); 6SQ8 (AMP); 6H1B (AMP); 7YWK (AMP); 5N81 (8Q2); 5N82 (8PZ)
**PhoQ**	1ID1 (ANP); 3CGY (RDC); 6BLK (ATP); 1BXD (ANP); 3SL3 (ATP)
**LesA**	3H2K (BOG)
AGS: Phosphothiophosphoric Acid-Adenylate Ester; ATP: Adenosine-5'-Triphosphate; ANP: Phosphoaminophosphonic Acid-Adenylate Ester; ACP: Phosphomethylphosphonic Acid Adenylate Ester; ADP: Adenosine-5'-Diphosphate; D4F: [[(2R,3S,4R,5R)-5-(6-Aminopurin-9-Yl)-3,4-Bis(Oxidanyl)Oxolan-2-Yl]Methoxy-Oxidanyl-Phosphoryl] (4S)-2-(6-Oxidanyl-1,3-Benzothiazol-2-Yl)-4,5-Dihydro-1,3-Thiazole-4-Carboxylate; SLU: 5'-O-[N-(Dehydroluciferyl)-Sulfamoyl] Adenosine;8Q2 :[(2~{R},3~{S},4~{R},5~{R})-5-(6-Aminopurin-9-Yl)-3,4-Bis(Oxidanyl)Oxolan-2-Yl]Methyl ~{N}-[(3~{S})-3-Azanyl-3-(4-Prop-2-Ynoxyphenyl)Propanoyl]Sulfamate; 8PZ: [(2~{R},3~{S},4~{R},5~{R})-5-(6-Aminopurin-9-Yl)-3,4-Bis(Oxidanyl)Oxolan-2-Yl]Methyl ~{N}-[(3~{S})-3-Azanyl-3-Phenyl-Propanoyl]Sulfamate; RDC: Radicicol; BOG: octyl beta-D-glucopyranoside.	

S2 Validation of predicted protein structural models

To validate the structural model generated by computational modeling of the selected protein targets for *X. fastidiosa* listed in Table 1, we used the following protocol. UniProt FASTA sequences (224) were submitted to BLAST (225) to identify PDB-available homologs with ≥40% identity and ≥80% coverage. Domain annotations were retrieved via INTERPRO (226), considering only structure-focused databases: Gene3D (227), SUPERFAMILY (228), FunFAM (229), Pfam (230), and CDD (231). Structural alignments were performed in PyMOL (The PyMOL Molecular Graphics System, Version 3.0 Schrödinger, LLC), comparing each AlphaFold domain to its PDB counterparts.

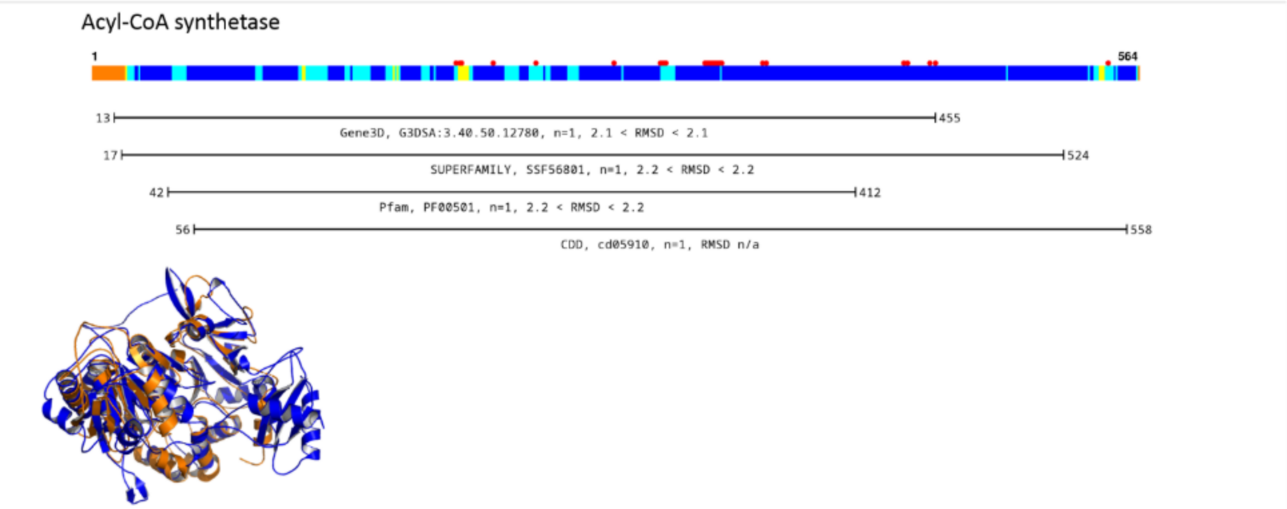

ChiA

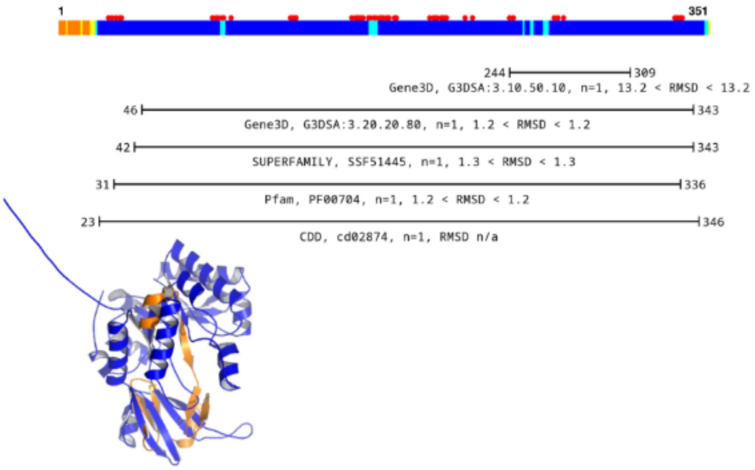

FimA

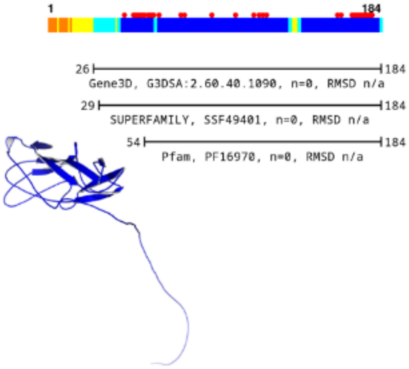

FimF

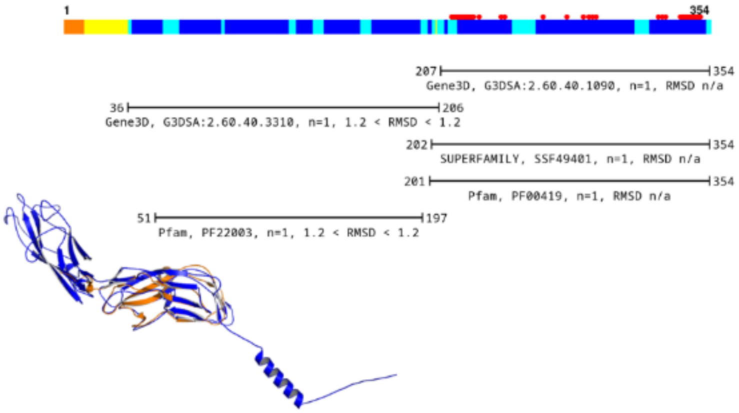

LesA

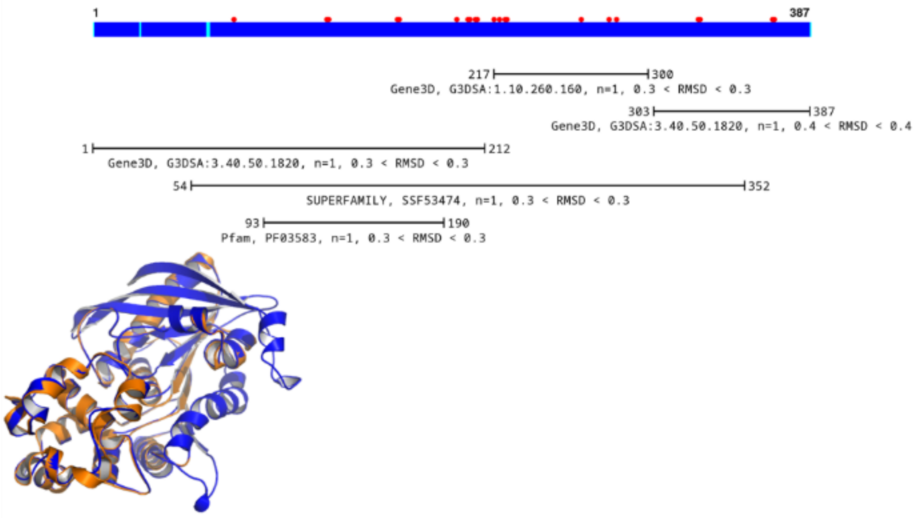
1
387
217 300
Gene3D, G3DSA:1.10.260.160, n=1, 0.3 < RMSD < 0.3
303 387
Gene3D, G3DSA:3.40.50.1820, n=1, 0.4 < RMSD < 0.4
1 212
Gene3D, G3DSA:3.40.50.1820, n=1, 0.3 < RMSD < 0.3
54 352
SUPERFAMILY, SSF53474, n=1, 0.3 < RMSD < 0.3
93 190
Pfam, PF03583, n=1, 0.3 < RMSD < 0.3

PhoQ

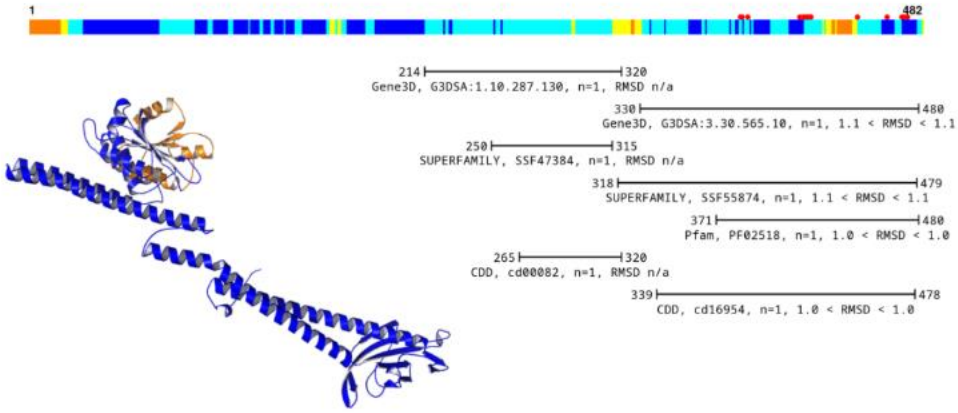
1
482
214 320
Gene3D, G3DSA:1.10.287.130, n=1, RMSD n/a
330 480
Gene3D, G3DSA:3.30.565.10, n=1, 1.1 < RMSD < 1.1
250 315
SUPERFAMILY, SSF47384, n=1, RMSD n/a
318 479
SUPERFAMILY, SSF55874, n=1, 1.1 < RMSD < 1.1
371 480
Pfam, PF02518, n=1, 1.0 < RMSD < 1.0
265 320
CDD, cd00082, n=1, RMSD n/a
339 478
CDD, cd16954, n=1, 1.0 < RMSD < 1.0

RpfB

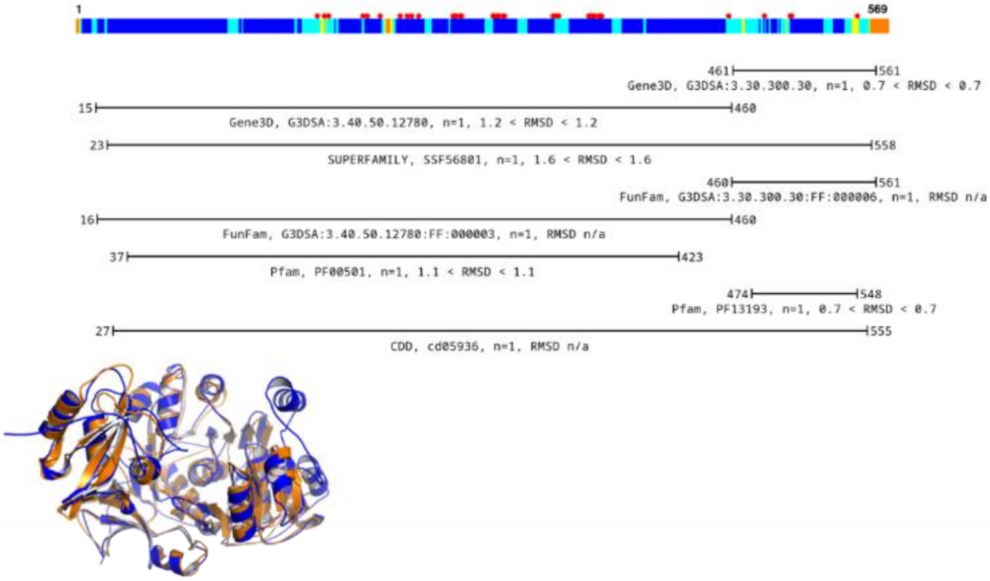
1
569
461 561
Gene3D, G3DSA:3.30.300.30, n=1, 0.7 < RMSD < 0.7
15 460
Gene3D, G3DSA:3.40.50.12780, n=1, 1.2 < RMSD < 1.2
23 558
SUPERFAMILY, SSF56801, n=1, 1.6 < RMSD < 1.6
460 561
FunFam, G3DSA:3.30.300.30:FF:000006, n=1, RMSD n/a
16 460
FunFam, G3DSA:3.40.50.12780:FF:000003, n=1, RMSD n/a
37 423
Pfam, PF00501, n=1, 1.1 < RMSD < 1.1
474 548
Pfam, PF13193, n=1, 0.7 < RMSD < 0.7
27 555
CDD, cd05936, n=1, RMSD n/a

XatA

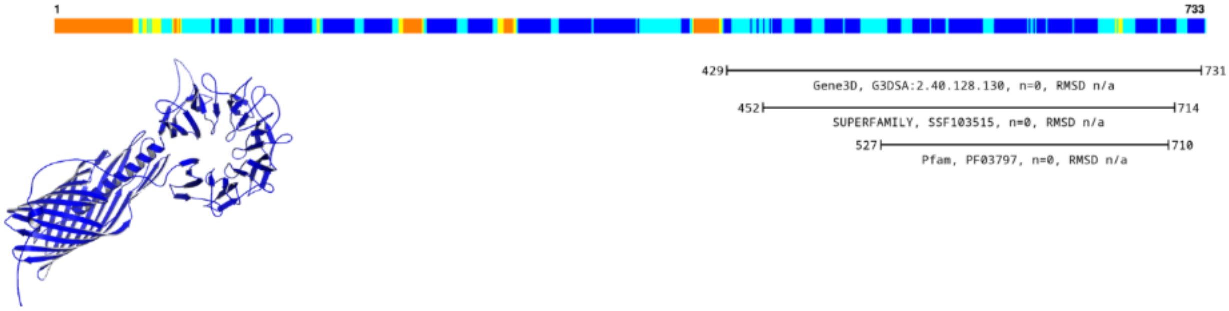
1
733
429 731
Gene3D, G3DSA:2.40.128.130, n=0, RMSD n/a
452 714
SUPERFAMILY, SSF103515, n=0, RMSD n/a
527 710
Pfam, PF03797, n=0, RMSD n/a

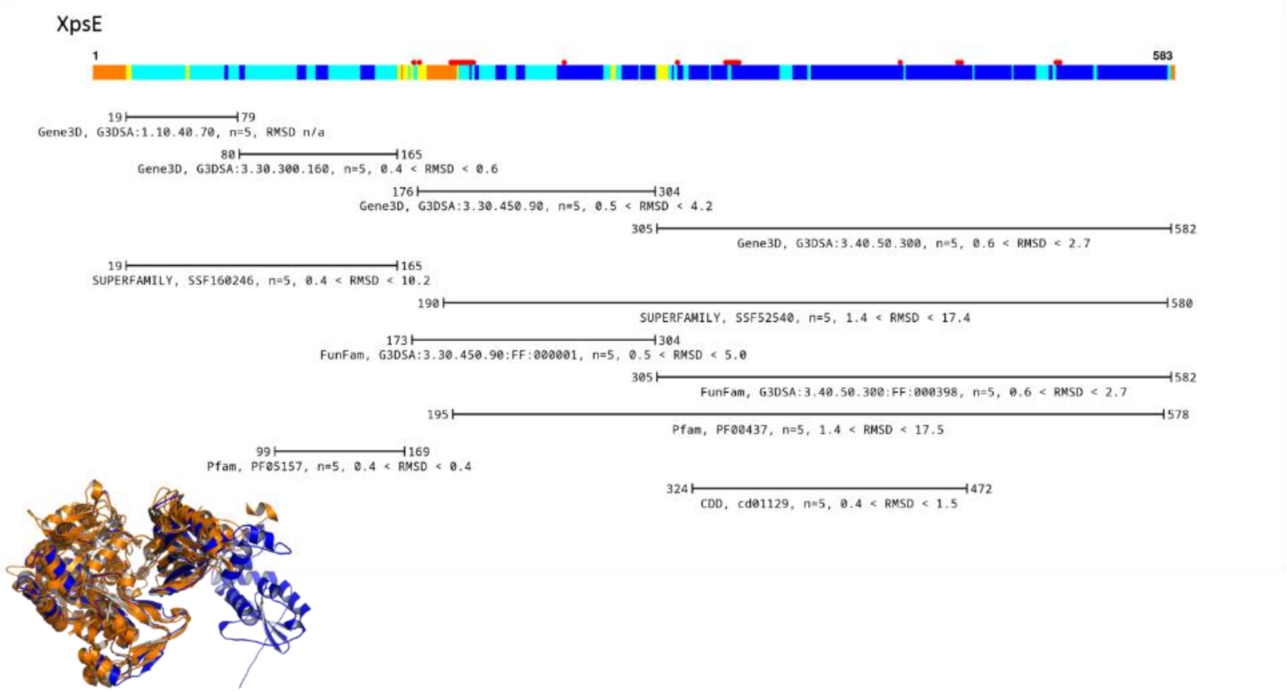

Figure S1. Validation of the AlphaFold-predicted structures for selected virulence factor targets. For each target, the following elements are shown: a color bar representing the AlphaFold confidence scores (pLDDT), with the following color scheme — blue for pLDDT > 90, cyan for 70 < pLDDT ≤ 90, yellow for 50 < pLDDT ≤ 70, and orange for pLDDT ≤ 50 — and black bars indicating the domains identified by InterPro. For each domain, the corresponding structure-focused database, domain code, number of homologous sequences, and the minimum and maximum RMSD values obtained from structural alignment between the target and BLAST-identified homologs are reported. The numbers at the beginning and end of both the color and black bars indicate the respective start and end residues. Red dots above the pLDDT bar identify the residues for draggability predictions. Additionally, the best structural superposition between the selected factor (shown in blue) and the most similar BLAST-derived domain (shown in orange) is displayed.

A

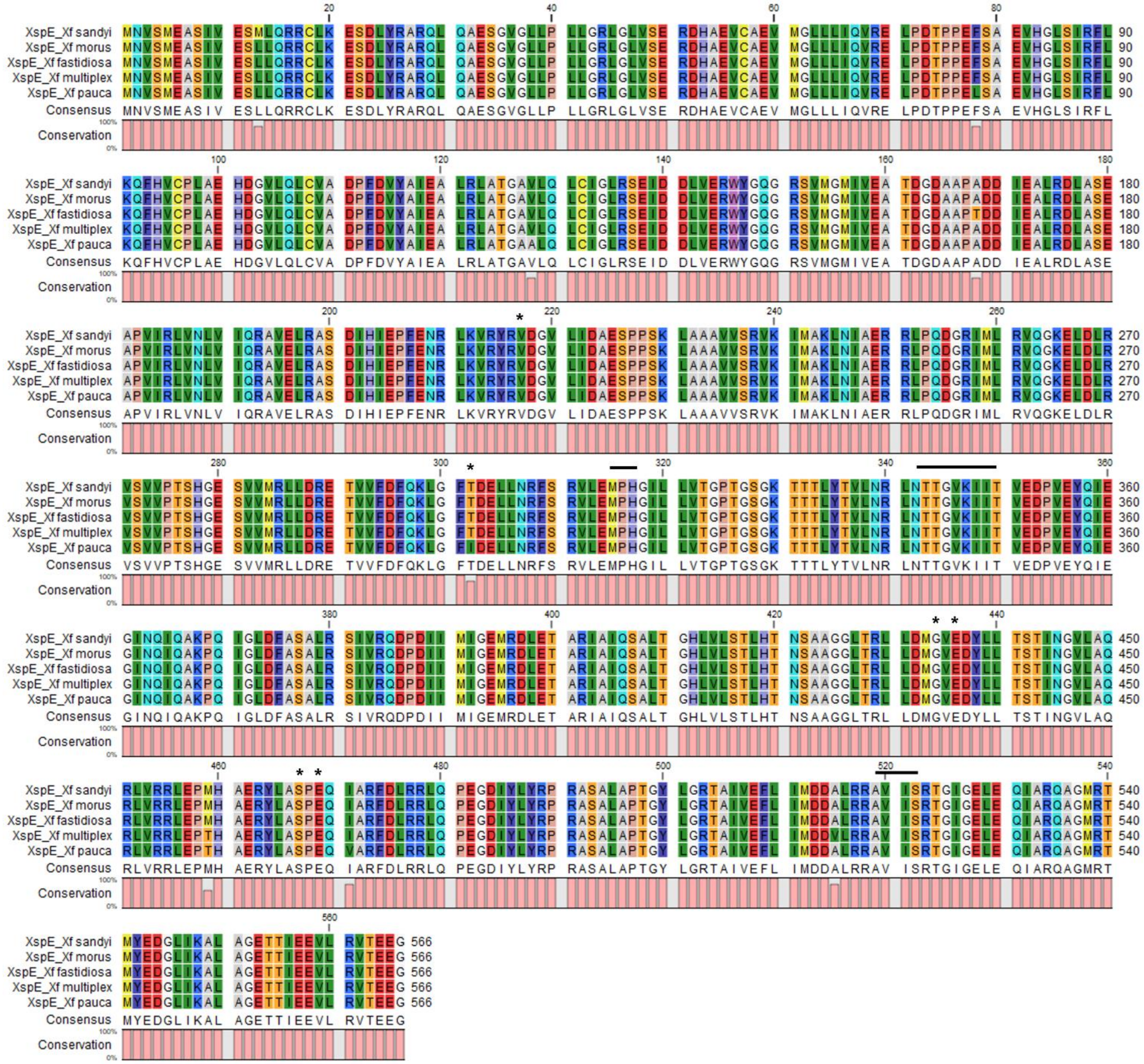
XspE_Xf sandyi
XspE_Xf morus
XspE_Xf fastidiosa
XspE_Xf multiplex
XspE_Xf pauca
Consensus
Conservation
MNVSMEASIV ESLLQRRCLK ESDLYRARQL QAESGVGLLP LLGRLGLVSE RDHAEVCAEV MGLLLIQVRE LPDTPPEFSA EVHGLSIRFL 90
KQFHVCPLAE HDGVLQLCVA DPFDVYAIEA LRLATGAVLQ LCIGLRSEID DLVERWYGQG RSVMGMIVEA TDGDAAPADD IEALRDLASE 180
APVIRLVNLV IQRAVELRAS DIHIEPFENR LKVRYRVDGV LIDAESPPSK LAAAVVSRVK IMAKLNIAER RLPQDGRIML RVQGKELDLR 270
VSVVPTSHGE SVVMRLLDRE TVVFDFQKLG FTDELLNRFS RVLEMPHGIL LVTGPTGSGK TTTLYTVLNR LNTTGVKIIT VEDPVEYQIE 360
GINQIQAKPQ IGLDFASALR SIVRQDPDII MIGEMRDLET ARIAIQSALT GHLVLSTLHT NSAAGGLTRL LDMGVEDYLL TSTINGVLAQ 450
RLVRRLEPMH AERYLASPEQ IARFDLRRLQ PEGDIYLYRP RASALAPTGY LGRTAIVEFL IMDDALRRAV ISRTGIGELE QIARQAGMRT 540
MYEDGLIKAL AGETTIEEVL RVTEEG 566

B

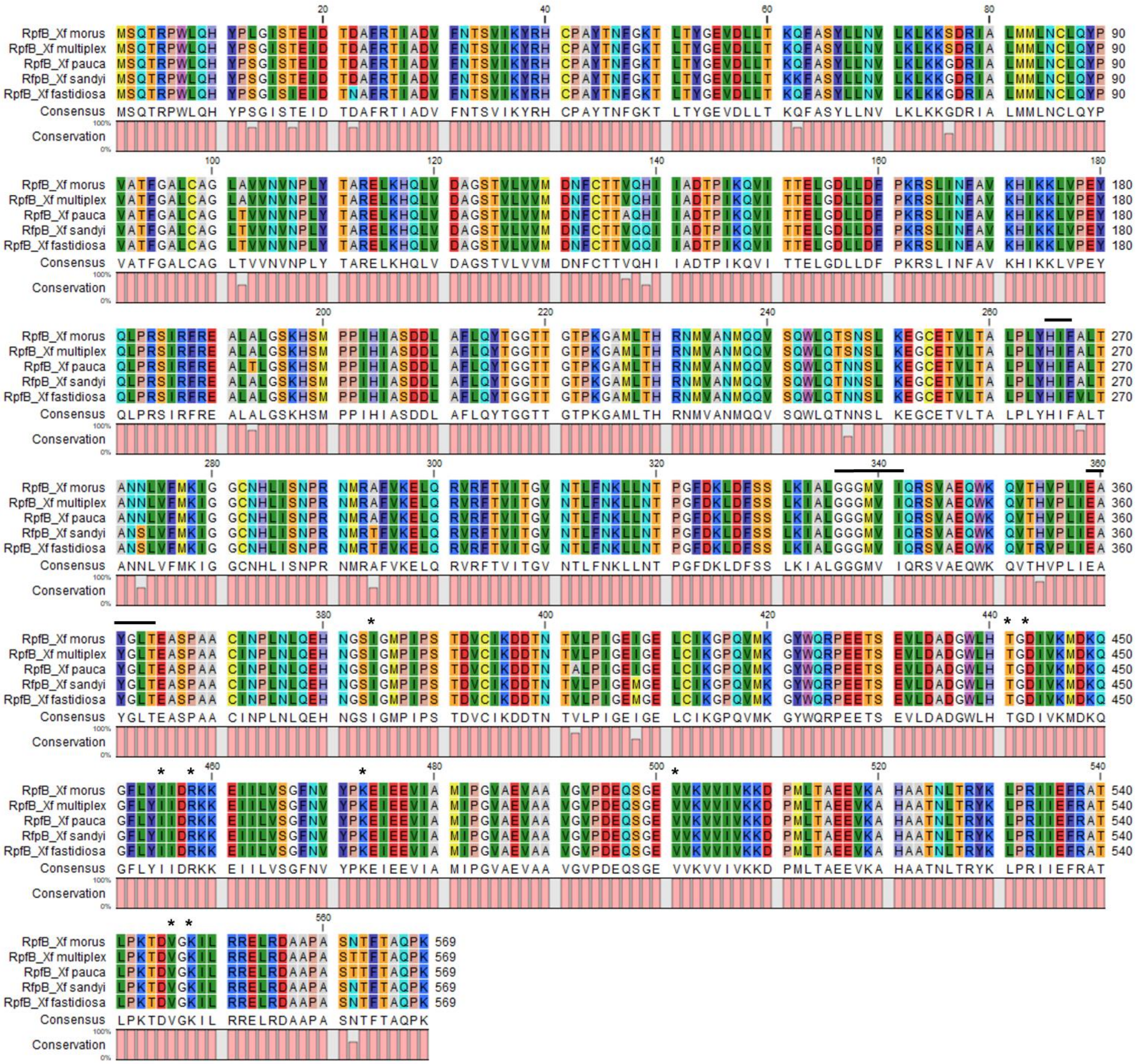

RpfB_Xf morus
RpfB_Xf multiplex
RpfB_Xf pauca
RfpB_Xf sandyi
RpfB_Xf fastidiosa
Consensus MSQTRPWLQH YPSGISTEID TDAFRTIADV FNTSVIKYRH CPAYTNFGKT LTYGEVDLLT KQFASYLLNV LKLKKGDRIA LMMLNCLQYP
Conservation
Consensus VATFGALCAG LTVVNVNPLY TARELKHQLV DAGSTVLVVM DNFCTTVQHI IADTPIKQVI TTELGDLLDF PKRSLINFAV KHIKKLVPEY
Consensus QLPRSIRFRE ALALGSKHSM PPIHIASDDL AFLQYTGGTT GTPKGAMLTH RNMVANMQQV SQWLQTNNSL KEGCETVLTA LPLYHIFALT
Consensus ANNLVFMKIG GCNHLISNPR NMRAFVKELQ RVRFTVITGV NTLFNKLLNT PGFDKLDFSS LKIALGGGMV IQRSVAEQWK QVTHVPLIEA
Consensus YGLTEASPAA CINPLNLQEH NGSIGMPIPS TDVCIKDDTN TVLPIGEIGE LCIKGPQVMK GYWQRPEETS EVLDADGWLH TGDIVKMDKQ
Consensus GFLYIIDRKK EIILVSGFNV YPKEIEEVIA MIPGVAEVAA VGVPDEQSGE VVKVVIVKKD PMLTAEEVKA HAATNLTRYK LPRIIEFRAT
Consensus LPKTDVGKIL RRELRDAAPA SNTFTAQPK

C

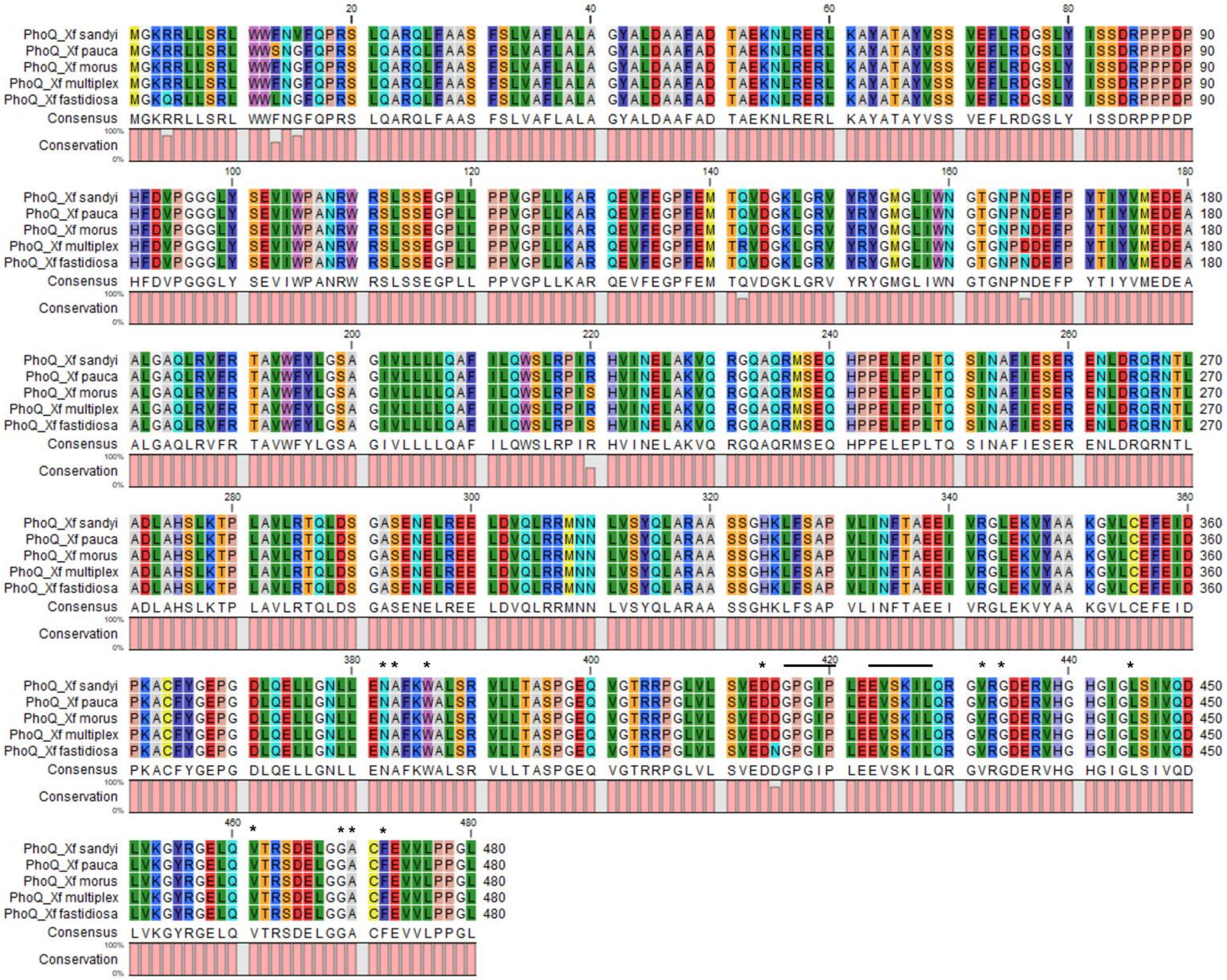
20 40 60 80
PhoQ_Xf sandyi MGKRRLLSRL WWFNVFQPRS LQARQLFAAS FSLVAFLALA GYALDAAFAD TAEKNLRERL KAYATAYVSS VEFLRDGSLY ISSDRPPPDP 90
PhoQ_Xf pauca MGKRRLLSRL WWSNGFQPRS LQARQLFAAS FSLVAFLALA GYALDAAFAD TAEKNLRERL KAYATAYVSS VEFLRDGSLY ISSDRPPPDP 90
PhoQ_Xf morus MGKRRLLSRL WWFNGFQPRS LQARQLFAAS FSLVAFLALA GYALDAAFAD TAEKNLRERL KAYATAYVSS VEFLRDGSLY ISSDRPPPDP 90
PhoQ_Xf multiplex MGKRRLLSRL WWFNGFQPRS LQARQLFAAS FSLVAFLALA GYALDAAFAD TAEKNLRERL KAYATAYVSS VEFLRDGSLY ISSDRPPPDP 90
PhoQ_Xf fastidiosa MGKQRLLSRL WWLNGFQPRS LQARQLFAAS FSLVAFLALA GYALDAAFAD TAEKNLRERL KAYATAYVSS VEFLRDGSLY ISSDRPPPDP 90
Consensus MGKRRLLSRL WWFNGFQPRS LQARQLFAAS FSLVAFLALA GYALDAAFAD TAEKNLRERL KAYATAYVSS VEFLRDGSLY ISSDRPPPDP
Conservation 100% 0%
100 120 140 160 180
PhoQ_Xf sandyi HFDVPGGGLY SEVIWPANRW RSLSSEGPLL PPVGPLLKAR QEVFEGPFEM TQVDGKLGRV YRYGMGLIWN GTGNPNDEFP YTIYVMEDEA 180
PhoQ_Xf pauca HFDVPGGGLY SEVIWPANRW RSLSSEGPLL PPVGPLLKAR QEVFEGPFEM TQVDGKLGRV YRYGMGLIWN GTGNPNDEFP YTIYVMEDEA 180
PhoQ_Xf morus HFDVPGGGLY SEVIWPANRW RSLSSEGPLL PPVGPLLKAR QEVFEGPFEM TQVDGKLGRV YRYGMGLIWN GTGNPNDEFP YTIYVMEDEA 180
PhoQ_Xf multiplex HFDVPGGGLY SEVIWPANRW RSLSSEGPLL PPVGPLLKAR QEVFEGPFEM TRVDGKLGRV YRYGMGLIWN GTGNPDDEFP YTIYVMEDEA 180
PhoQ_Xf fastidiosa HFDVPGGGLY SEVIWPANRW RSLSSEGPLL PPVGPLLKAR QEVFEGPFEM TQVDGKLGRV YRYGMGLIWN GTGNPNDEFP YTIYVMEDEA 180
Consensus HFDVPGGGLY SEVIWPANRW RSLSSEGPLL PPVGPLLKAR QEVFEGPFEM TQVDGKLGRV YRYGMGLIWN GTGNPNDEFP YTIYVMEDEA
Conservation 100% 0%
200 220 240 260
PhoQ_Xf sandyi ALGAQLRVFR TAVWFYLGSA GIVLLLLQAF ILQWSLRPIR HVINELAKVQ RGQAQRMSEQ HPPELEPLTQ SINAFIESER ENLDRQRNTL 270
PhoQ_Xf pauca ALGAQLRVFR TAVWFYLGSA GIVLLLLQAF ILQWSLRPIR HVINELAKVQ RGQAQRMSEQ HPPELEPLTQ SINAFIESER ENLDRQRNTL 270
PhoQ_Xf morus ALGAQLRVFR TAVWFYLGSA GIVLLLLQAF ILQWSLRPIS HVINELAKVQ RGQAQRMSEQ HPPELEPLTQ SINAFIESER ENLDRQRNTL 270
PhoQ_Xf multiplex ALGAQLRVFR TAVWFYLGSA GIVLLLLQAF ILQWSLRPIR HVINELAKVQ RGQAQRMSEQ HPPELEPLTQ SINAFIESER ENLDRQRNTL 270
PhoQ_Xf fastidiosa ALGAQLRVFR TAVWFYLGSA GIVLLLLQAF ILQWSLRPIS HVINELAKVQ RGQAQRMSEQ HPPELEPLTQ SINAFIESER ENLDRQRNTL 270
Consensus ALGAQLRVFR TAVWFYLGSA GIVLLLLQAF ILQWSLRPIR HVINELAKVQ RGQAQRMSEQ HPPELEPLTQ SINAFIESER ENLDRQRNTL
Conservation 100% 0%
280 300 320 340 360
PhoQ_Xf sandyi ADLAHSLKTP LAVLRTQLDS GASENELREE LDVQLRRMNN LVSYQLARAA SSGHKLFSAP VLINFTAEEI VRGLEKVYAA KGVLCEFEID 360
PhoQ_Xf pauca ADLAHSLKTP LAVLRTQLDS GASENELREE LDVQLRRMNN LVSYQLARAA SSGHKLFSAP VLINFTAEEI VRGLEKVYAA KGVLCEFEID 360
PhoQ_Xf morus ADLAHSLKTP LAVLRTQLDS GASENELREE LDVQLRRMNN LVSYQLARAA SSGHKLFSAP VLINFTAEEI VRGLEKVYAA KGVLCEFEID 360
PhoQ_Xf multiplex ADLAHSLKTP LAVLRTQLDS GASENELREE LDVQLRRMNN LVSYQLARAA SSGHKLFSAP VLINFTAEEI VRGLEKVYAA KGVLCEFEID 360
PhoQ_Xf fastidiosa ADLAHSLKTP LAVLRTQLDS GASENELREE LDVQLRRMNN LVSYQLARAA SSGHKLFSAP VLINFTAEEI VRGLEKVYAA KGVLCEFEID 360
Consensus ADLAHSLKTP LAVLRTQLDS GASENELREE LDVQLRRMNN LVSYQLARAA SSGHKLFSAP VLINFTAEEI VRGLEKVYAA KGVLCEFEID
Conservation 100% 0%
380 * * * 400 * 420 * * 440 *
PhoQ_Xf sandyi PKACFYGEPG DLQELLGNLL ENAFKWALSR VLLTASPGEQ VGTRRPGLVL SVEDDGPGIP LEEVSKILQR GVRGDERVHG HGIGLSIVQD 450
PhoQ_Xf pauca PKACFYGEPG DLQELLGNLL ENAFKWALSR VLLTASPGEQ VGTRRPGLVL SVEDDGPGIP LEEVSKILQR GVRGDERVHG HGIGLSIVQD 450
PhoQ_Xf morus PKACFYGEPG DLQELLGNLL ENAFKWALSR VLLTASPGEQ VGTRRPGLVL SVEDDGPGIP LEEVSKILQR GVRGDERVHG HGIGLSIVQD 450
PhoQ_Xf multiplex PKACFYGEPG DLQELLGNLL ENAFKWALSR VLLTASPGEQ VGTRRPGLVL SVEDDGPGIP LEEVSKILQR GVRGDERVHG HGIGLSIVQD 450
PhoQ_Xf fastidiosa PKACFYGEPG DLQELLGNLL ENAFKWALSR VLLTASPGEQ VGTRRPGLVL SVEDNGPGIP LEEVSKILQR GVRGDERVHG HGIGLSIVQD 450
Consensus PKACFYGEPG DLQELLGNLL ENAFKWALSR VLLTASPGEQ VGTRRPGLVL SVEDDGPGIP LEEVSKILQR GVRGDERVHG HGIGLSIVQD
Conservation 100% 0%
460 * * * * 480
PhoQ_Xf sandyi LVKGYRGELQ VTRSDELGGA CFEVVLPPGL 480
PhoQ_Xf pauca LVKGYRGELQ VTRSDELGGA CFEVVLPPGL 480
PhoQ_Xf morus LVKGYRGELQ VTRSDELGGA CFEVVLPPGL 480
PhoQ_Xf multiplex LVKGYRGELQ VTRSDELGGA CFEVVLPPGL 480
PhoQ_Xf fastidiosa LVKGYRGELQ VTRSDELGGA CFEVVLPPGL 480
Consensus LVKGYRGELQ VTRSDELGGA CFEVVLPPGL
Conservation 100% 0%

D

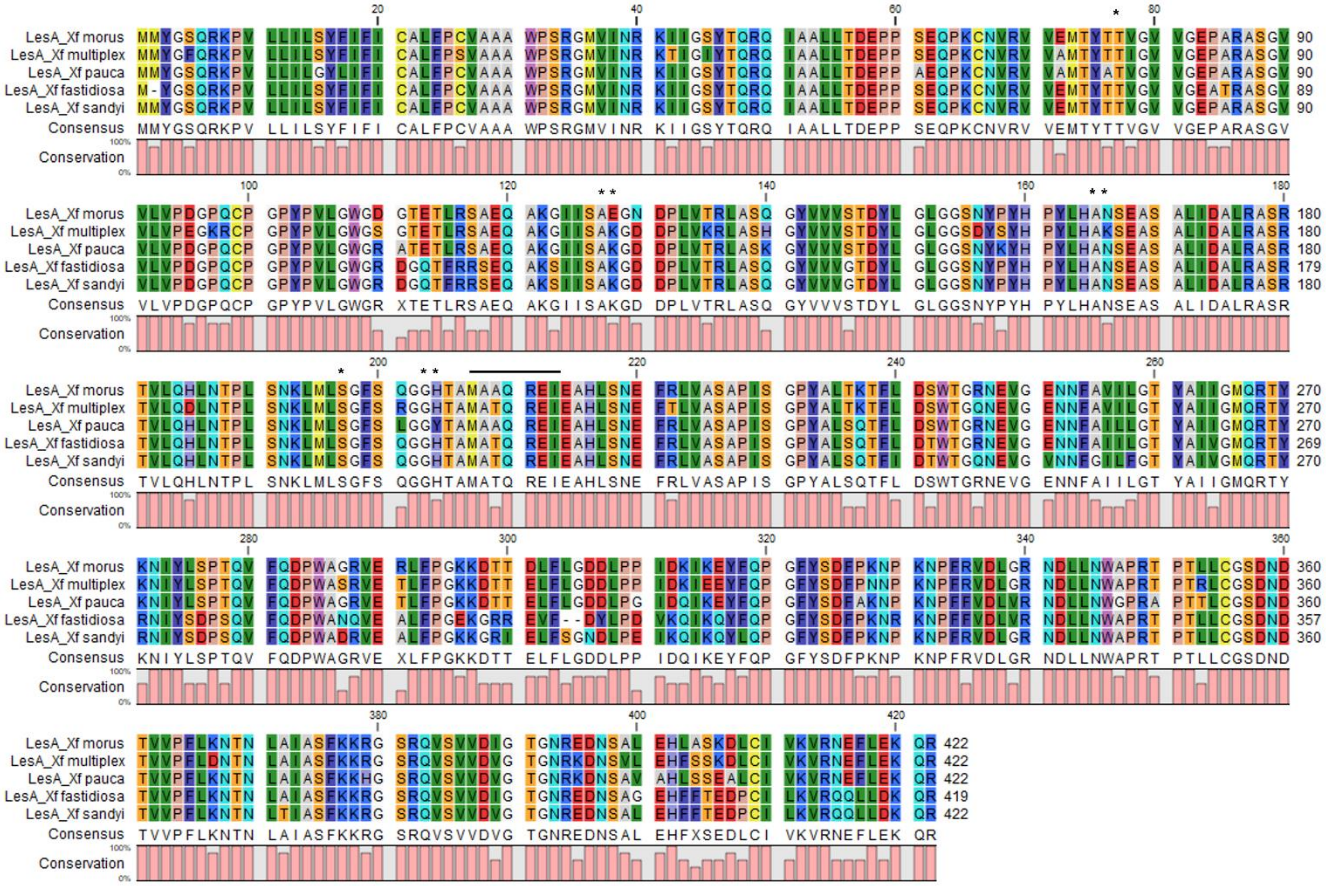

Figure S2

Multiple sequence alignment of the selected protein target XpsE (A), RpfB (B), PhoQ (C) and LesA (D) from different *X. fastidiosa* subspecies: *fastidiosa*, *multiplex*, *sandyi*, *pauca* and *morus.* Black stars and lines indicate the predicted key binding sites residues (Table 2). The alignment was performed with Geneious® 10.2.3 and CLC Sequence viewer (Qiagen). The details about strains, genomes, and genes IDs were described in Table S2.

Table S2. Subspecies, strains, genomes, and genes IDs used for alignment of amino acids in Figure S3.

Subspecie	Strain	Genome ID	XpsE (GspE)	RpfB	PhoQ	LesA
morus	Riv16	NZ_CP090316.1	WP_020852927.1	WP_040123293.1	WP_038210684.1	WP_038227880.1
sandyi	CO33	NZ_CP135132.1	WP_057682491.1	WP_057683078.1	WP_200904596.1	WP_057682733.1
fastidiosa	Temecula1	NC_004556.1	WP_012382531.1	WP_011097553.1	WP_014607440.1	WP_394326516.1
multiplex	TOS4	GCA_007713905.1	WP_004083688.1	WP_004085319.1	WP_021358376.1	WP_145510268.1
pauca	De Donno	NZ_CP020870.1	WP_046419592.1	WP_046417883.1	WP_171817042.1	WP_046417961.1
